# Clinical correlates of circulating cell-free DNA tumor fraction

**DOI:** 10.1371/journal.pone.0256436

**Published:** 2021-08-25

**Authors:** Joerg Bredno, Jafi Lipson, Oliver Venn, Alexander M. Aravanis, Arash Jamshidi

**Affiliations:** GRAIL, Inc., Menlo Park, California, United States of America; IRCCS Ospedale Policlinico San Martino, Genova, Italy, ITALY

## Abstract

**Background:**

Oncology applications of cell-free DNA analysis are often limited by the amount of circulating tumor DNA and the fraction of cell-free DNA derived from tumor cells in a blood sample. This circulating tumor fraction varies widely between individuals and cancer types. Clinical factors that influence tumor fraction have not been completely elucidated.

**Methods and findings:**

Circulating tumor fraction was determined for breast, lung, and colorectal cancer participant samples in the first substudy of the Circulating Cell-free Genome Atlas study (CCGA; NCT02889978; multi-cancer early detection test development) and was related to tumor and patient characteristics. Linear models were created to determine the influence of tumor size combined with mitotic or metabolic activity (as tumor mitotic volume or excessive lesion glycolysis, respectively), histologic type, histologic grade, and lymph node status on tumor fraction. For breast and lung cancer, tumor mitotic volume and excessive lesion glycolysis (primary lesion volume scaled by percentage positive for Ki-67 or PET standardized uptake value minus 1.0, respectively) were the only statistically significant covariates. For colorectal cancer, the surface area of tumors invading beyond the subserosa was the only significant covariate. The models were validated with cases from the second CCGA substudy and show that these clinical correlates of circulating tumor fraction can predict and explain the performance of a multi-cancer early detection test.

**Conclusions:**

Prognostic clinical variables, including mitotic or metabolic activity and depth of invasion, were identified as correlates of circulating tumor DNA by linear models that relate clinical covariates to tumor fraction. The identified correlates indicate that faster growing tumors have higher tumor fractions. Early cancer detection from assays that analyze cell-free DNA is determined by circulating tumor fraction. Results support that early detection is particularly sensitive for faster growing, aggressive tumors with high mortality, many of which have no available screening today.

## Introduction

An inherent limitation of circulating tumor DNA (ctDNA) analysis techniques is the proportion of cell-free DNA (cfDNA) that is derived from tumor cells in a blood sample [1]. This proportion, or circulating tumor fraction (cTF), is usually very low when tumors are small and localized, but increases as they grow and metastasize [2–4]. In addition, cTF varies widely between individuals, cancer types [3], and clinical stage [5]. These variations have important implications for a wide range of clinical applications of ctDNA-based assays, including multi-cancer early detection (MCED) tests [6–9] and minimal residual disease detection [10–13]; however, the factors that influence cTF have not been completely elucidated.

**Fig 1:**
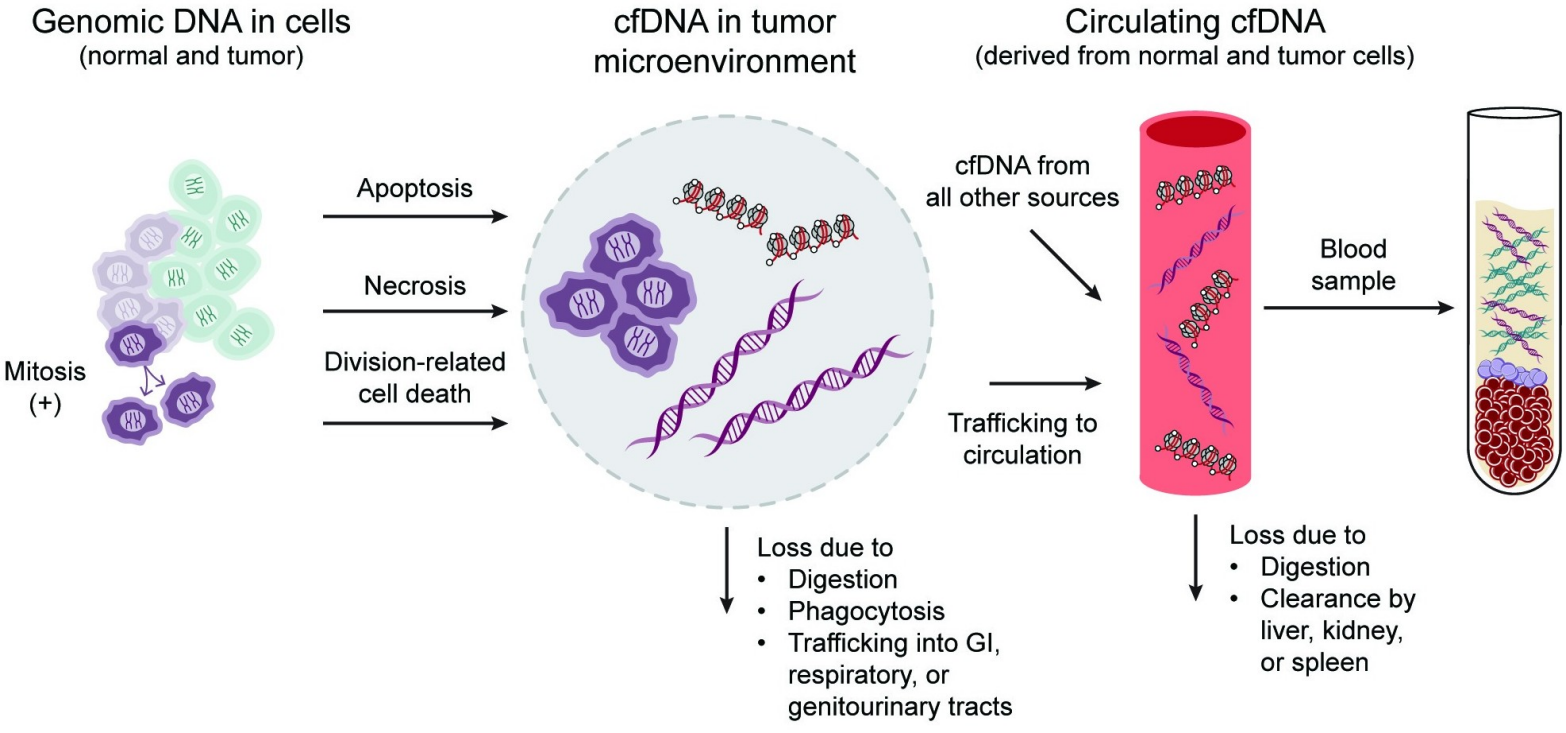
Depiction of origin and fates of circulating tumor DNA relative to cell-free DNA. Origin and fates of cfDNA influence the amount of ctDNA in a blood sample. Both normal cells (light green) and tumor cells (light purple) can shed DNA during cell death (e.g., by apoptosis or necrosis) [14–16]. In cancer, tumor cell mitoses and tumor growth increase the amount of DNA that can be shed into the tumor microenvironment (TME) [17]. In the TME, cfDNA has several fates: it may be digested [18, 19]; phagocytosed [17, 21]; lost into the lumen of the gastrointestinal, pulmonary, or genitourinary tract [22]; or trafficked into circulation where it is pooled with cfDNA from other cells in the body [21, 23]. After entering circulation, cfDNA is subject to further digestion or clearance in the liver, kidney, or spleen [23]. As a result, cfDNA in a blood sample is composed of tumor and normal cell DNA that is shed by dying cells and has not been removed by various clearance mechanisms. cfDNA: cell-free DNA, ctDNA: circulating tumor DNA, TME: tumor microenvironment.

Underlying this knowledge gap is an incomplete understanding of the mechanisms responsible for cfDNA shedding first into the tumor microenvironment, access from there to the circulatory system, and elimination, which determine the observed cTF in a blood sample (Fig 1) [14–23].

Although the general characteristics of this process are well established, detailed models of the mechanisms of cfDNA shedding and factors that influence the amount in circulation—and consequently, detectability and clinical utility—for different types or stages of tumors have yet to be described [14]. Current evidence suggests that the origin of ctDNA in a blood sample is biased towards tumor cells that are aggressive or susceptible to treatment, which lead to proliferation- or treatment-induced apoptosis and cfDNA shedding [14]. In addition, preliminary evidence suggests that high-mortality cancers in the bottom 10th percentile of 5-year survival rates in the United States (esophageal, gastric, hepatobiliary, lung, and pancreatic cancer) tend to have higher cTF across all stages than lower-mortality cancers (e.g., breast, prostate, and thyroid cancer) [5]. These observations suggest that ctDNA-based cancer screening tests may be more likely to detect lethal cancers, which could help reduce overdiagnosis and unnecessary interventions [24].

To better understand how cTF varies across tumor stages and affects blood-based cancer detection, we developed models of ctDNA shedding using clinical features of tumor biology beyond clinical stage (e.g., tumor size and mitotic or metabolic activity). Using breast, lung, and colorectal cancers, which have the highest incidence and mortality among all cancers in men and women in the United States [25], we identified several key correlates of ctDNA levels and biological tumor properties that can be generalized to other solid cancers.

## Methods

### Analysis overview

The Circulating Cell-free Genome Atlas (CCGA; NCT02889978) study is a prospective, multicenter, observational, case-control study with longitudinal follow-up to support the development of a plasma circulating cfDNA-based multi-cancer early detection test. The CCGA protocol and consent were reviewed and approved by the Institutional Review Board (IRB) or Independent Ethics Committee (IEC) for each of the 140 participating sites and the central IRB Western IRB (now WIRB Copernicus Group). The other IRBs/ethics boards were Hartford HealthCare IRB, Hartford, CT, US Oncology, Inc., IRB, The Woodlands, TX, Memorial Sloan Kettering Cancer Center Institutional Review Board/Privacy Board, New York, NY, University of Miami Institutional Review Board, Miami, FL, Mayo Clinic Institutional Review Board, Rochester, MN, Cleveland Clinic Foundation Institutional Review Board, Cleveland, OH, Avera Central Services IRB #3—Oncology IRB, Sioux Falls, SD, Lahey Clinic, Inc. Institutional Review Board, Burlington, MA, Biomedical Research Alliance of New York, Lake Success, NY, The Christ Hospital Institutional Review Board, Cincinnati, OH, University Health Network Research Ethics Board, Toronto, ON, Canada, Lehigh Valley Health Network’s Institutional Review Board, Allentown, PA, IntegReview Ethical Review Board, Austin, TX, and Dana-Farber Cancer Institute (DFCI) IRB, Boston, MA. IRBs provide oversight of the study throughout its duration. All participants were consented per regulatory requirements prior to participating in study-related activities and sample collection. In CCGA, blood samples were prospectively collected from participants with newly diagnosed untreated cancer and from participants without a diagnosis of cancer. Samples from the first [26] and second [9] CCGA substudy were used to develop and validate biophysical models (separately for breast, lung, and colorectal cancers) that identify clinical correlates of cTF.

**Fig 2:**
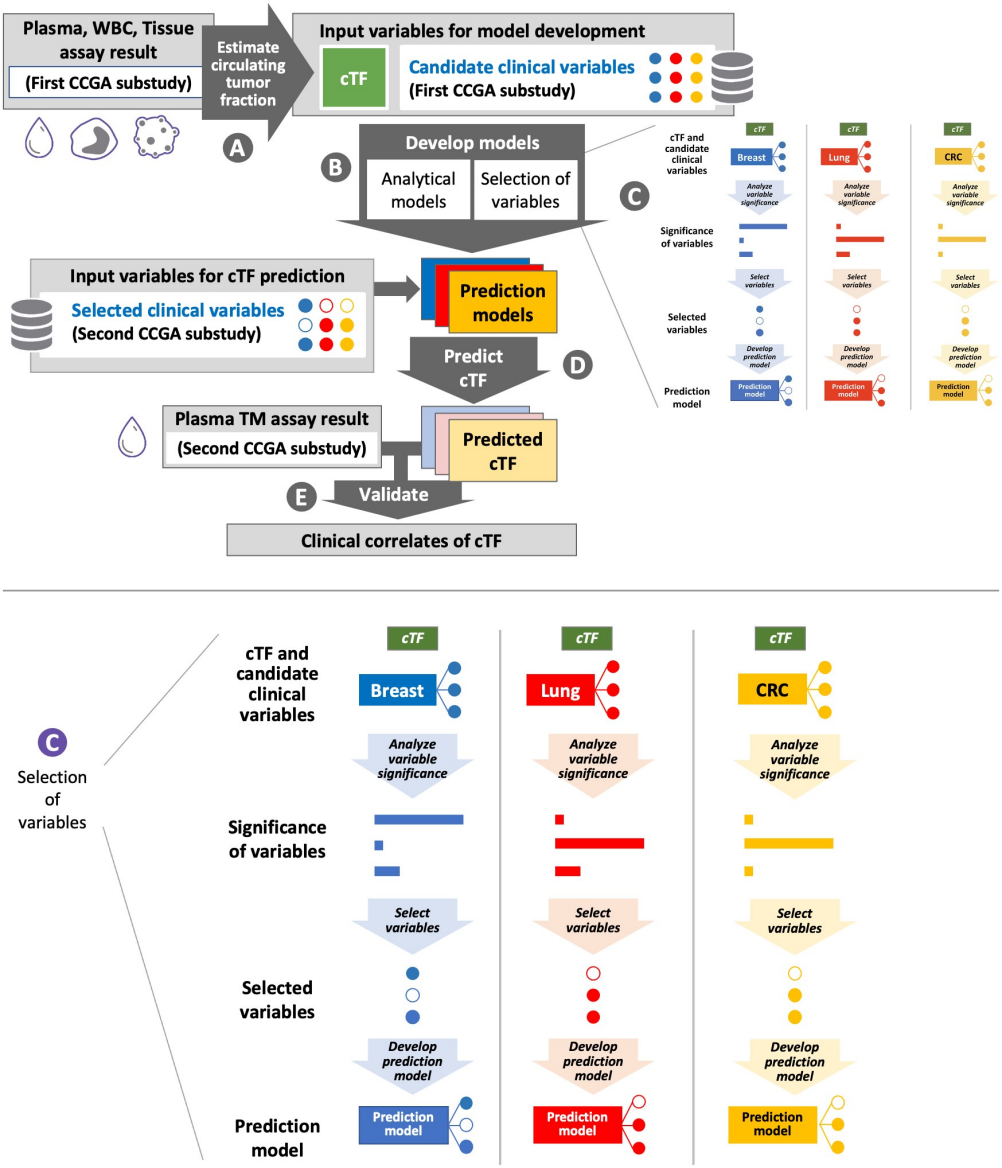
Flow diagram of data analysis. cTF was obtained from plasma, WBC, and tissue assay results (step A) and candidate clinical variables were input for model development (step B). During model development, candidate variables that contributed significantly to cTF were selected as correlates of tumor fraction (step C, insert shown enlarged). Filled and hollow dots depict variables selected or not selected, respectively. cTF was predicted using only selected clinical variables (step D) and was validated by comparison to plasma TM assay results (step E). CCGA: Circulating Cell-free Genome Atlas, cfDNA: cell-free DNA, CRC, colorectal cancer, cTF: Circulating tumor fraction, WBC: White blood cells, TM: Targeted Methylation.

Fig 2 depicts the analysis process. Briefly, paired plasma, white blood cells (WBC), and tissue samples from participants in the first CCGA substudy (for which whole genome sequencing [WGS], whole-genome bisulfite sequencing [WGBS], and targeted sequencing data were available) [26] were used to obtain estimated circulating tumor fraction cTF, (described below, Fig 2, step A). Independent of cTF estimation, candidate input clinical variables and covariates were identified for model development if they had been linked to ctDNA levels in previous publications [27–30], or as motivated by their involvement in the pathophysiological processes of ctDNA generation shown in Fig 1 [14–23]. Linear models were created that relate cTF to the candidate clinical variables for participants from the first CCGA substudy (Fig 2, step B). Variables were selected for inclusion in a prediction model if they significantly contribute to cTF (Fig 2, step C, insert shown enlarged at the bottom of the figure). These models then predicted cTF for participants in the second CCGA substudy using only selected clinical variables (Fig 2, step D). For validation, model-predicted cTF was compared to tumor detection from a plasma sample using the targeted methylation (TM) assay of the second CCGA substudy (Fig 2, step E). CCGA study design, WGBS, TM panel design and sequencing, sample collection, storage, accessioning and processing, determination of cTF and creation of a WGBS and a TM classifier for cancer detection have been previously presented [5, 9, 26].

### Circulating tumor fraction

cTF was determined from blood samples prospectively collected from participants with newly diagnosed untreated cancer from the first CCGA substudy. Genomic variants detected in a targeted sequencing assay of plasma cfDNA from these participants were compared to variants from a WGS assay of matched, macro-dissected formalin-fixed, paraffin-embedded tumor samples while using a white blood cell WGS assay to control for germline variants [5] (Fig 2, step A). For cases with no available tumor tissue, cTF was imputed using scores from a prototype classifier that was trained to detect cancer signals in WGBS data from plasma cfDNA [26]. This WGBS classifier score is computed from abnormally methylated cfDNA fragments in separate genomic regions and has previously been shown to track cTF measurements [5, 30] with an approximately sigmoid relationship of WGBS classifier score to log(cTF). Parameters of the sigmoid function and their 95% confidence intervals were estimated separately per tumor type. cTF was imputed by drawing parameters of this fitted curve randomly for each case from their estimated distributions and performing a lookup from classifier score to cTF.

For additional quantitative interpretation, the concentration of cfDNA in plasma was quantified using a High Sensitivity Large Fragment Analysis Kit (DNF-493, Fragment Analyzer, Agilent). The concentration was scaled by tumor fraction and an estimated total volume of whole blood from participant weight and height [31] assuming plasma volume as 55% of whole blood volume. This yielded the total mass of ctDNA in participants’ circulation and was scaled to genome equivalents (GE) using 6.5 pg / GE.

### Candidate clinical variable selection

Clinical data for this analysis were obtained from CCGA electronic case report forms and cancer-type specific data from pathology and radiology reports. The goal of each biophysical model is to quantitatively explain circulating tumor DNA using only a small subset of routinely available clinical features.

Analyses were limited to clinical stages I, II, and III because ctDNA levels increase strongly in the presence of distant metastases, especially when a highly vascularized organ like the liver is affected [13, 32] and because tumor fraction in stage IV cancers is higher [5] and more variable compared to stages I-III, which would significantly complicate the modeling.

Similarly, clinical stage was not selected as a covariate for the purpose of modeling, given that ctDNA levels increase with clinical stage with overlap between stages I, II, III [5, 27, 29, 30] and that the definition of clinical stage depends on the cancer type and takes multiple clinical variables and covariates into account [33] that modeling can test separately.

The selection of candidate clinical correlates and covariates was motivated by the goal to capture an absolute rate of cancer cell deaths taking the total number of tumor cells and their apoptotic and necrotic rates into account. Even when not accounting for cellularity, total tumor volume can serve as a measure for the total number of tumor cells. Total volume computations should further reflect tumor laterality and focality, and the size of each lesion, as all lesions individually contribute ctDNA. Abstracted clinical data provided 1D maximum size per lesion and information on multifocal or bilateral disease. Total volume of all primary lesions was computed assuming spherical lesions. Missing size information was only imputed for non-index tumor lesions when the index lesion size was reported. A ratio of index lesion to non-index lesion size was drawn randomly from the second and third quartile of this ratio for cases with the same cancer type and complete size information. This ratio was multiplied with the reported index lesion size to impute size of non-index lesions.

The presence of tumor-involved lymph nodes was confirmed either by pathology report or clinical node (N)-stage N1, N2, or N3. The absence was confirmed by N-stage N0 and confirmation that all examined lymph nodes in the pathology report were negative. In the absence of pathology report information and clinical N-stage, presence of tumor-involved lymph nodes was imputed from the clinical stage and assumed present for clinical stages III and IV.

Cell death rates(eg., using % positive of the immunohistochemistry marker cleaved caspase 3) are not routinely reported. Available clinical variables for mitotic or metabolic tumor activity were chosen for model creation assuming that tumor cells constantly outgrow tumor resources [18] with hypoxic apoptosis, mitotic catastrophe [34–36] or other forms of cell death closely following cell division [21, 37].

### Stepwise creation of biophysical models

Following selection of the candidate clinical variables, a linear analytical model was created to identify statistically significant (p-value < 0.05) clinical correlates of tumor fraction (Fig 2 Step C). Additionally, their relative importance to explain cTF was determined using R-squared (R^2^) partitioned by averaging over orders [38, 39]. Next, only the clinical variables identified to significantly contribute to cTF were selected as input to a linear prediction model (Fig 2, step C). Finally, a quantitative linear model for each cancer type was created to explain the total number of tumor-derived GEs in the patient body using the same selected clinical variables.

Analyses were performed in the statistical software program R version 3.6.0 with linear model fitting taken from the stats package and relative importance metrics computed with the relaimpo package in version 2.2.3. For validation, receiver operating characteristic (ROC) curves were created and analyzed using the pROC package in version 1.16.2 and the Wilcoxon rank-sum test was taken from the stats package.

### Breast cancer model motivation

The impact of different clinical features of breast cancer on cTF has been previously presented [27]. cTF increases with tumor (T) stage, N stage, hormone-receptor (HR) status, and percent of tumor nuclei positive for Ki-67 (%Ki-67), but not with human epidermal growth factor receptor 2 (HER2) status or histologic type. It has been shown for breast cancer that %Ki-67 positive for mitotic rate and % positive of cleaved caspase 3 for apoptotic rate are correlated [40], and %Ki-67 positive is frequently reported in breast cancer.

### Breast cancer model implementation

The basis of a biophysical model to predict cTF in breast cancer is the tumor mitotic volume (TMitV), which is the tumor volume multiplied by %Ki-67 positive. In CCGA, %Ki-67 positive was reported for breast cancer from participating sites. Breast cancer tumor tissue submitted for study purposes was additionally sent for Ki-67 staining and read-out to a CAP/CLIA (College of American Pathologists/Clinical Laboratory Improvement Amendments)-certified laboratory for cases from sites that allowed such an additional read-out per protocol.

TMitV is intended to capture ctDNA from all primary tumor foci. More ctDNA is expected from tumor-involved lymph nodes and also from distant metastasis which are not considered here. Tumor-involved lymph nodes widely vary in number, tumor content and location, which is challenging to capture in clinical variables reported in a multi-site study. As a modeling variable, presence of tumor-involved lymph nodes (yes/no) was used due to less complete data for the number of tumor-involved lymph nodes. Histologic type (ductal or lobular carcinoma), tumor grade, and hormone receptor status (positive if a case was positive for progesterone or estrogen receptor overexpression) were tested as additional, independent predictors of cTF in the linear analysis model.

### Lung cancer model motivation

Different clinical features of lung cancer that impact cTF have been presented [28, 29]. For non-small lung cancer (NSCLC), in univariate analysis the presence of necrosis, lymph node involvement, lymphovascular invasion, tumor size, %Ki-67 positive, and a histologic type other than adenocarcinoma increased cTF. In a multivariable analysis, non-adenocarcinoma subtype, high Ki-67, and lymphovascular invasion were individual predictors for detection of ctDNA in plasma [28]. Furthermore, cTF increases with (18)F fluorodeoxyglucose (FDG) uptake on Positron Emission Tomography—Computed Tomography (PET/CT) for NSCLC [28, 29] and small cell lung cancer [11]. While %Ki-67 positive is not routinely reported for lung cancer, FDG PET/CT is often available. The standardized uptake value (SUV) from FDG PET is correlated to tumor growth [41, 42] and %Ki-67 positive in breast [43, 44] and lung cancers [45].

### Lung cancer model implementation

FDG PET SUV was obtained by abstraction of PET/CT radiology reports. For lung cancers, prediction of ctDNA from all primary tumor lesions was based on the newly defined excessive lesion glycolysis (ELG), which is the volume integral of FDG PET SUV over all lesions after subtracting 1.0 from the SUV value. Normal tissue is expected to have an SUV close to 1.0 and to not create many cfDNA fragments, while tumor tissue has SUV > 1.0. ELG is closely related to total lesion glycolysis (TLG), which is the volume integral of SUV. Due to data availability in the CCGA study, ELG computation is simplified to volume scaled by a single SUV_max_—1.0. In previous studies, TLG was shown to correlate with ctDNA [28], but not with overall cfDNA levels [46]. Histologic type (adenocarcinoma, squamous cell carcinoma, small cell carcinoma), presence of tumor-involved lymph nodes, and histologic grade were tested as additional independent predictors of cTF.

### Colorectal cancer (CRC) model motivation

Previously, tumor surface area (TSA) and depth of microinvasion have been presented as correlates of cTF for colorectal adenocarcinomas [30]. In contrast to lung, breast, and many other solid cancers, colon cancer size is often measured after resection with the specimen spread from the original curved colon wall onto a flat surface. A surrogate measure for the total number of tumor cells is therefore the tumor surface area of all primary tumor foci together. Markers of cell death or proliferation like cleaved caspase 3, Ki-67, or FDG PET SUV are not frequently reported in CRC. Instead, while fewer cases are available for model creation and validation than for breast and lung cancers, we use CRC to show a different clinical correlate of cTFCTF. A candidate covariate captures how tumor vascularization and trafficking of DNA fragments released by tumor cells affects ctDNA levels. The depth of microinvasion (invaded tissue layers beyond the epithelial lining at the inside of the colon lumen) is routinely reported as it contributes to T stage in CRC [33]. Tumor DNA from apoptotic tumor cells can enter the colon lumen or the circulation [47]. Depth of microinvasion or Tstage therefore inform whether tumor-derived DNA can enter the circulation.

### CRC model implementation

The biophysical model for CRC therefore uses the TSA individually scaled by a shedding factor that depends on depth of microinvasion. Levels of ctDNA are again expected to increase with tumor size and the number of tumor cells, even though tumor size does not contribute to clinical staging in CRC. The analysis for CRC was limited to the dominant histologic type adenocarcinoma and only presence of tumor-involved lymph nodes and histologic grade were tested as additional independent correlates of tumor fraction.

### Model validation

For model validation, model predictions for cTF were generated for plasma cfDNA samples of participants in the second CCGA substudy (Fig 2, Step D). These samples were subjected to a TM assay with an independently trained machine-learning classifier for cancer detection and prediction of signal of origin for samples with detected cancer signal [9]. The clinical validation of a further refined TM assay and classifiers optimized for screening has recently been completed for a case-control study [48]. Preliminary results from a prospective cohort study evaluating clinical implementation of the MCED test have also been reported [49]. For the validation of the models presented in this paper, cTF predicted by a biophysical model was compared to cancer detected or not detected on an independent patient cohort and assay (Fig 2, Step E) to further relate cTF to early cancer detection test performance. Receiver operating characteristic (ROC) curves were created to test if model-predicted cTF can explain the behavior of the cancer detection test. Separately for each cancer type, a one-sided Wilcoxon test determined if our hypothesis that detected cancers have higher cTF and that cTF can be predicted from few clinical covariates held in the validation cohort.

## Results

**Fig 3:**
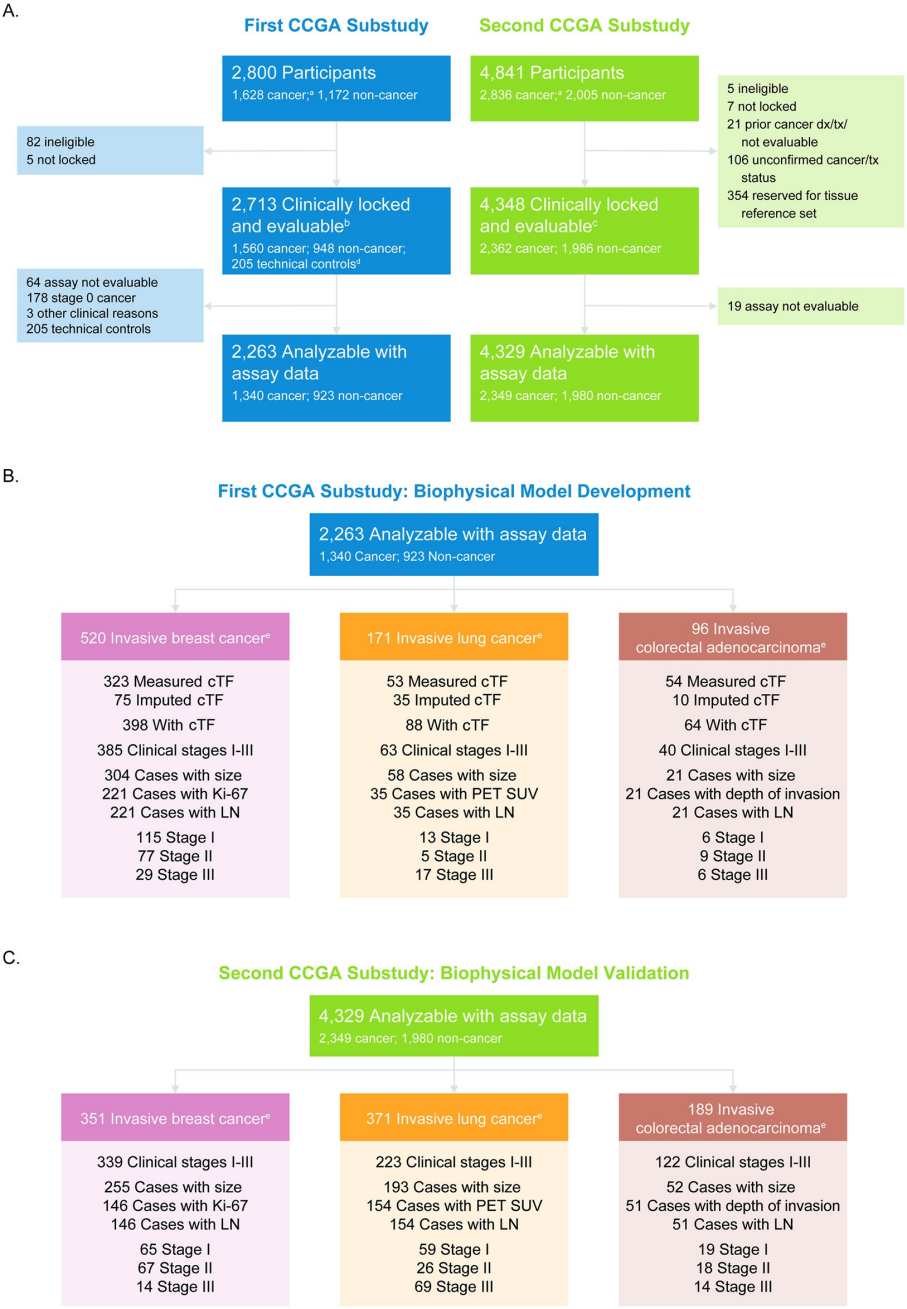
CONSORT diagram. (A) CONSORT diagram depicting the number of clinically evaluable cases with evaluable assay results for model generation from the first CCGA substudy (left) and model validation from the second CCGA substudy (right). (B) Cases available for model development from the first CCGA substudy and (C) cases available for model validation from the second CCGA substudy for breast (left), lung (center), and colorectal cancers (right). Cases were filtered by availability of clinical data (size of primary tumor, Ki-67 for breast cancer, PET SUV for lung cancer, depth of microinvasion for colorectal cancer, respectively, and presence of tumor-involved lymph nodes) for modeling and available ground truth or imputed tumor fraction. cTF: Circulating Tumor fraction, LN: information on number of tumor-involved lymph nodes, PET SUV, positron emission tomography standardized uptake value. ^a^At enrollment, prior to confirmation of cancer status. ^b^By First CCGA substudy definition. ^c^By Second CCGA substudy definition. ^d^Non-smoking participants under the age of 35. ^e^Confirmed cancer status.

For breast, lung, and colorectal cancer types, biophysical models were generated using cases from the first CCGA substudy and validated using cases from the second CCGA substudy (Fig 3A). For breast cancer, the number of cases used to generate and validate the model, respectively, were 221 and 146 (Fig 3B and 3C, left); for lung, the breakdown was 35 and 154 (Fig 3B and 3C, center); and for colorectal it was 21 and 51 (Fig 3B and 3C, right). Fig 3B and 3C also demonstrate, per cancer type, the breakdown of cases by available cTF measurements, available clinical covariates, and stage for model development and validation, respectively.

### Breast cancer

**Fig 4:**
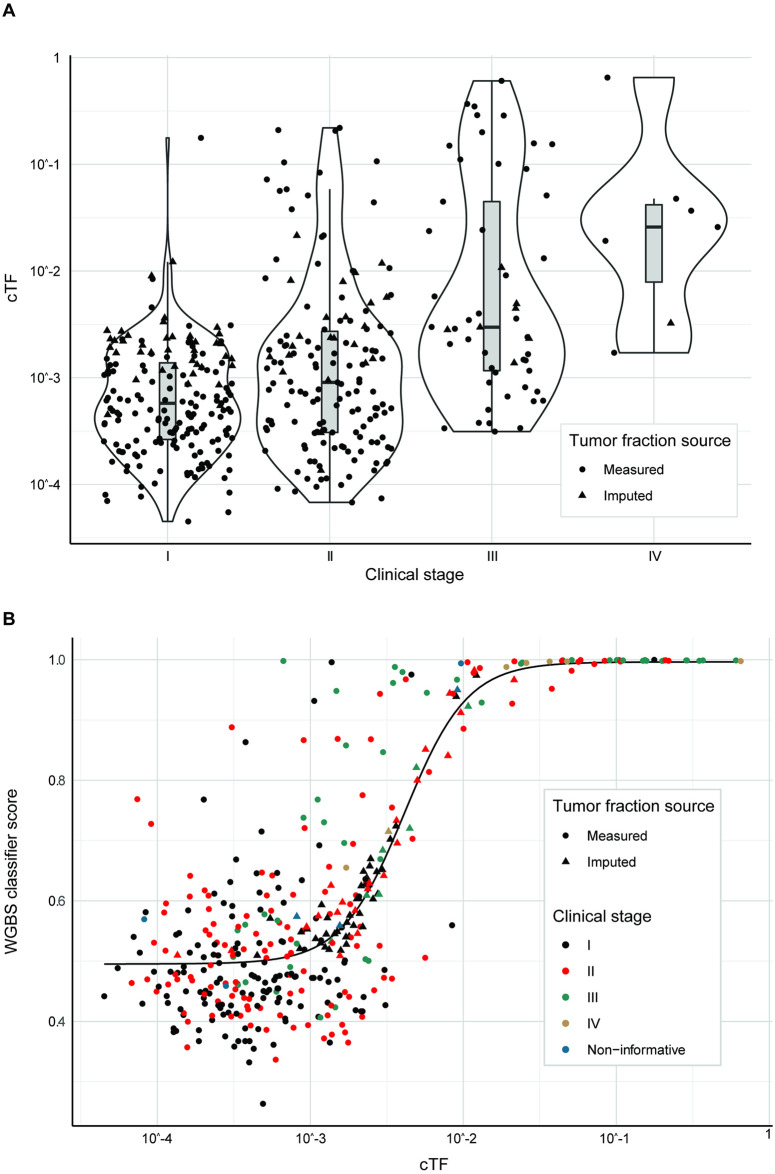
Breast cancer cTF by clinical stage and relation to WGBS classifier score. (A) cTF by clinical stage for breast cancer. (B) WGBS classifier score [26] by cTF for breast cancer. Samples are colored by clinical stage and samples with imputed cTF are shown as triangles. cTF: Circulating Tumor fraction, WGBS: Whole-genome bisulfite sequencing. Non-informative: Confirmed invasive cancer with insufficient clinical information to determine stage.

A total of 221 breast cancer cases (115 stage I, 77 stage II, 29 stage III) had cTF and sufficient clinical information to develop the biophysical model. 40 cases had cTF imputed from a WGBS classifier score, 17 cases had an imputed size for a non-index lesion, and lymph-node status was derived from clinical stage alone for 5 cases. The distribution of measured and imputed cTF for breast cancer cases were plotted by stage (Fig 4A), and confirm that, while cTF increases with stage in general, the distribution has strong overlap [27]. Fig 4B demonstrates that the WGBS prototype classifier scores [26] increased with tumor fraction, and the fitted sigmoid function used for cTF imputation. Additional cTF estimates (triangles) were imputed for cases with valid WGBS assay results but without a matched tissue sample. cTF distribution is shown for stage IV because these cases were used for imputation of cTF, however, only stage I-III cases were used in subsequent modeling (as described in the Methods section).

Table 1 shows the analytical model for breast cancer that determines the clinical variables that have statistically significant correlation with ctDNA. Only TMitV was identified to significantly contribute to cTF with a p-value <0.05, accounting for 45% of explained variability. Lymph node status, histologic grade, and HR-status accounted for 20%, 19% and 13%, respectively, while not statistically significant together with TMitV. Lobular or ductal histology did not appear to have a strong effect on cTF beyond TMitV.

**Table 1:** Analytical model for breast cancer.

Variable	Estimate	p-value	Relative importance
(Intercept)	0.0065	0.6601	NA
TMitV	2.85x10^−7^/mm^3^	**0.0075**	0.451
Lymph node status	0.0144	0.1100	0.201
Hormone receptor status	–0.0064	0.2624	0.134
Histologic grade (1 vs. 3)	0.0069	0.2561	0.187
Invasive lobular carcinoma	–0.0015	0.9372	0.010
Invasive ductal carcinoma	0.0027	0.8559	0.016

NA: not applicable; TMitV: Tumor mitotic volume.

**Fig 5:**
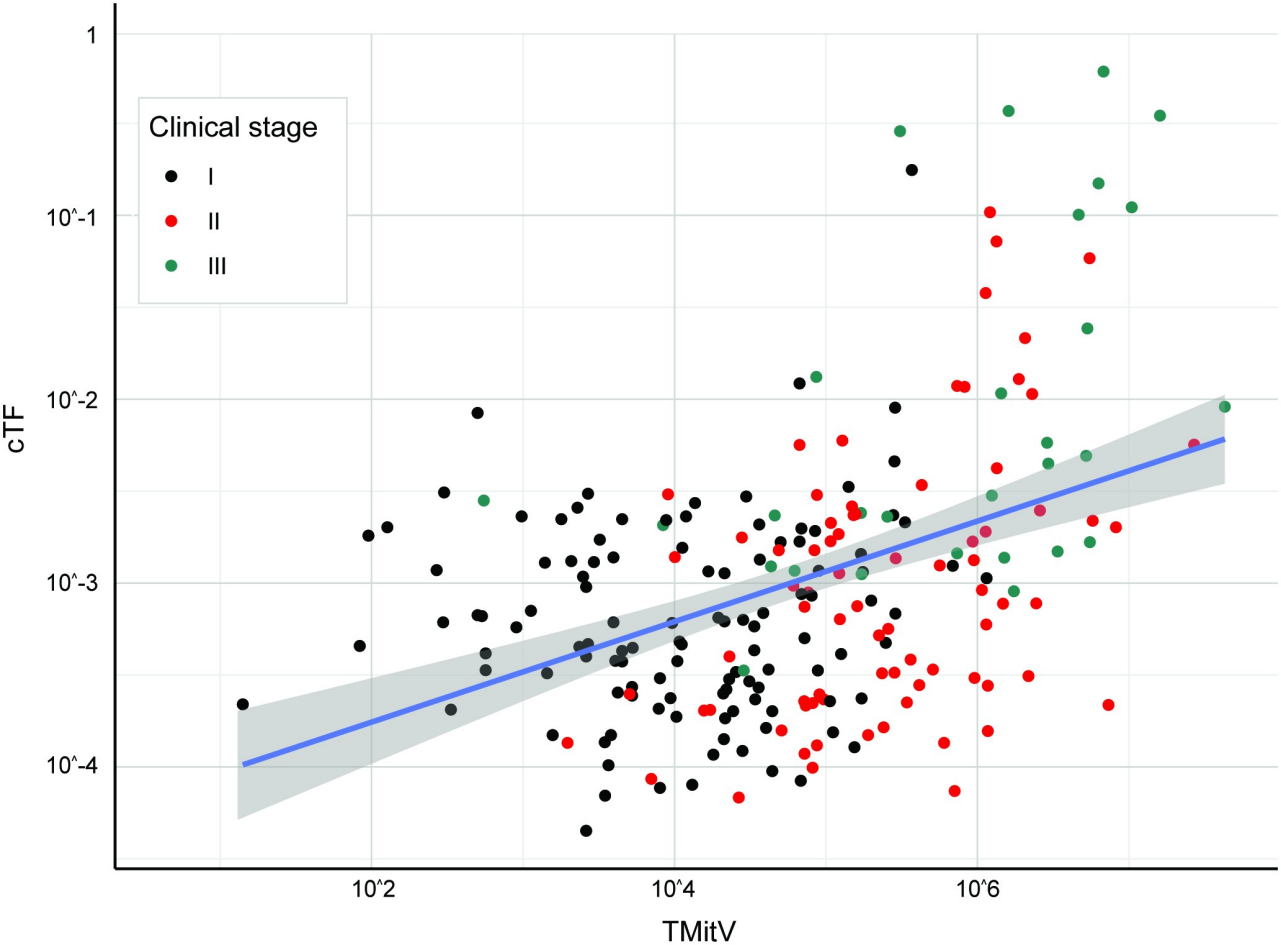
cTF increases with breast cancer tumor mitotic volume. cTF increased with TMitV (in mm^3^) for breast cancer. Black, red, and green dots depict samples from stages I, II, and II, respectively (Model development and validation was limited to clinical stages I-III). Linear fit and 95% confidence intervals are shown in blue and gray, respectively. cTF: Circulating cfDNA tumor fraction, TMitV: Tumor mitotic volume.

**Fig 6:**
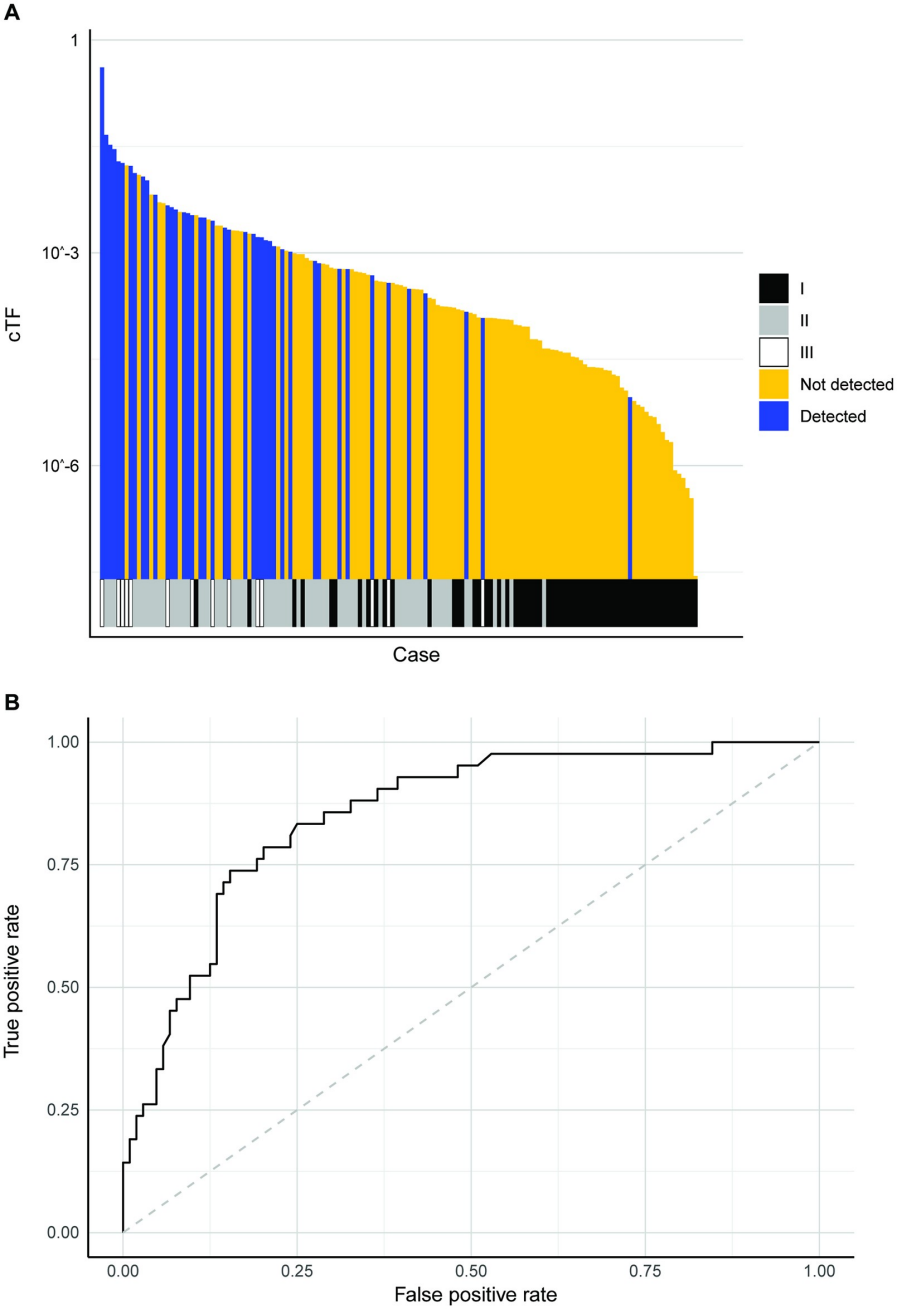
Breast cancer model validation. (A) Detection by TM assay for breast cancer cases sorted by TMitV. Clinical stage is shown at the bottom. (B) ROC to predict breast cancer detection by TM assay using TMitV. cTF: Circulating tumor fraction, ROC: Receiver operating characteristic curve, TM: Targeted methylation, TMitV: Tumor mitotic volume.

The linear prediction model was created using TMitV (Fig 5). 146 breast cancer cases from the CCGA2 substudy with TM results were available for model validation (1/65 detected in stage I, 28/67 detected in stage II, 13/14 detected in stage III). 9 cases had an imputed size for a non-index lesion. The prediction model separated detected and undetected cases beyond clinical stage (Fig 6A, Fig A in S1 Text, Wilcoxon rank-sum p-value <0.001) and can explain breast cancer detection with a TM assay with an area under curve (AUC) of 0.853 (95% CI 0.788–0.919) (Fig 6B).

The corresponding quantitative model estimated that each mm^3^ of mitotically active primary tumor volume adds 9.8 genome equivalents to the circulation of a breast cancer patient (p = 5.1x10^-6^).

### Lung cancer

**Fig 7:**
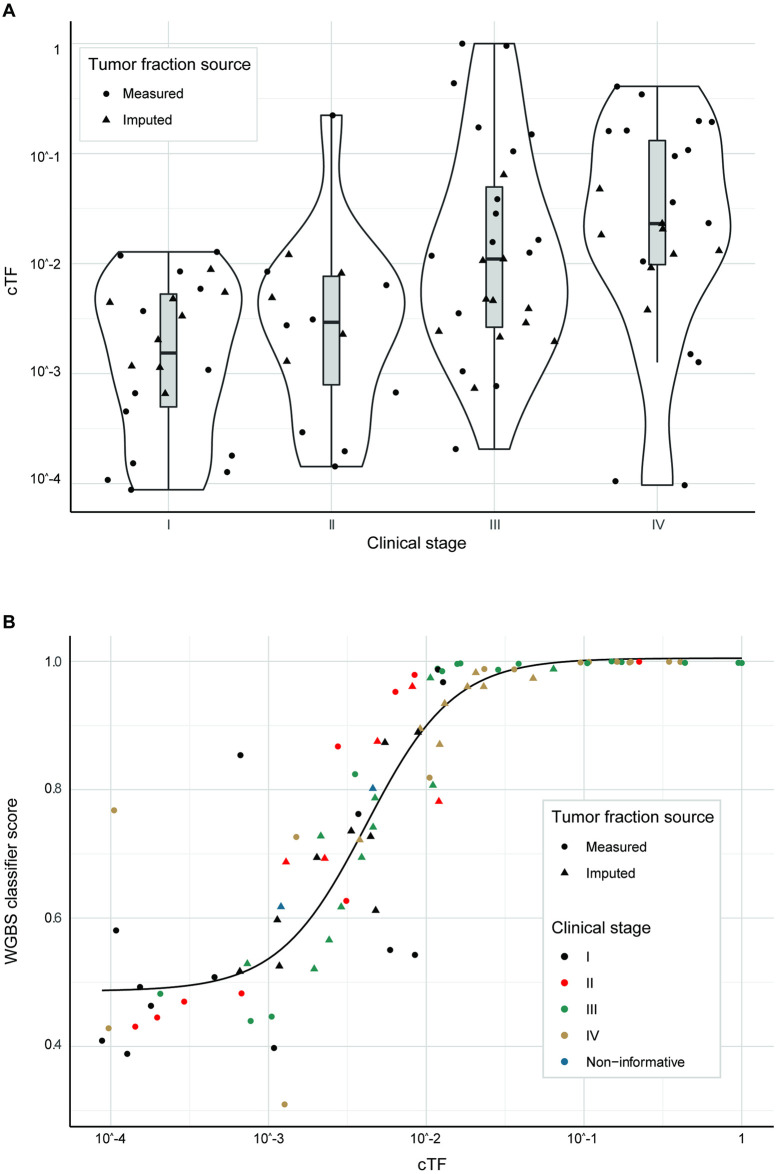
Lung cancer cTF by clinical stage and relation to WGBS classifier score. (A) cTF by clinical stage for lung cancer. (B) WGBS classifier score by cTF for lung cancer. Samples are colored by clinical stage and samples with imputed cTF are shown as triangles. cTF: Circulating Tumor fraction, WGBS: Whole-genome bisulfite sequencing. Non-informative: Confirmed invasive cancer with insufficient clinical information to determine stage.

For lung cancer, 35 cases (13 stage I, 5 stage II, 17 stage III) had cTF and sufficient clinical information to generate analysis, prediction, and quantitative models for ctDNA. 13 cases had cTF imputed from a WGBS classifier score and lymph-node status was derived from clinical stage alone for 1 case. Fig 7A shows the distribution of cTF for lung cancer cases for clinical stages I-IV and overlap especially between stages I and II. Fig 7B shows how WGBS prototype classifier scores increase with tumor fraction and again the matched sigmoid function and imputed cTF estimates.

In the analytical model for lung cancer, only ELG was identified to significantly contribute to cTF, accounting for 81% of explained variability (Table 2). While not statistically significant, histologic grade and presence of tumor-involved lymph nodes accounted for 14% and 3%, respectively. The histologic types adenocarcinoma, squamous cell carcinoma, and small cell lung cancer histology did not affect cTF beyond ELG. Tumor volume and metabolic activity measured by glycolysis accounts for previously published differences between lung cancer types [28, 29, 50].

**Table 2:** Analytical model for lung cancer.

Variable	Estimate	p-value	Relative importance
(Intercept)	0.0154	0.8697	NA
ELG	62.3x10^-9^/mm^3^	**< 0.001**	0.813
Lymph node status	0.0018	0.9749	0.029
Histologic grade (1 vs. 3)	0.0390	0.4492	0.138
Adenocarcinoma	–0.0036	0.9689	0.004
Squamous cell carcinoma	–0.0572	0.5604	0.010
Small cell lung cancer	0.0492	0.6830	0.006

ELG: Excessive lesion glycolysis; NA: not applicable.

**Fig 8:**
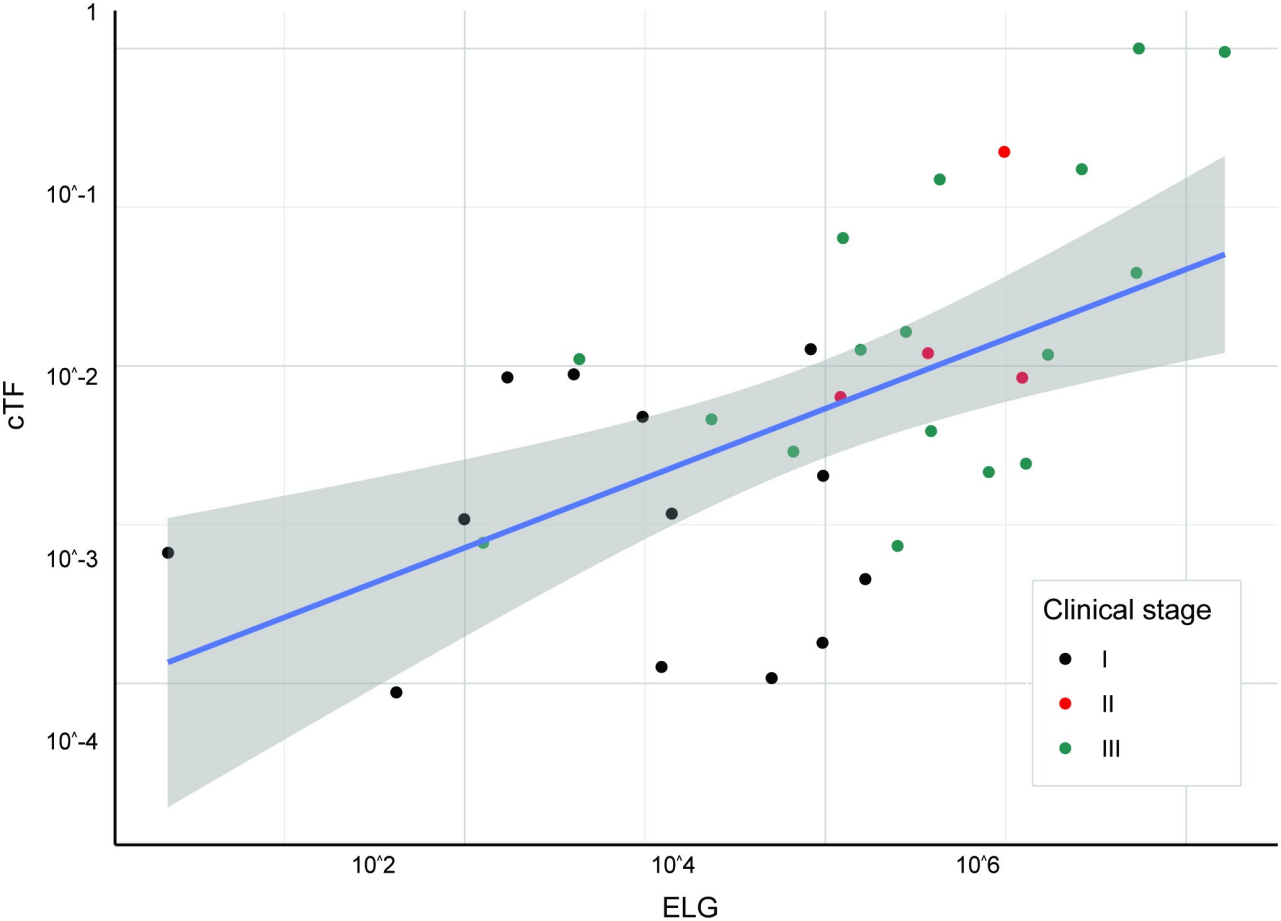
cTF increases with lung cancer excessive lesion glycolysis. **cTF** increases with ELG (in mm^3^) for lung cancer. Black, red, and green dots depict samples from stages I, II, and II, respectively (Model development and validation was limited to clinical stages I-III). Linear fit and 95% confidence intervals are shown in blue and gray, respectively. cTF: Circulating tumor fraction, ELG: Excessive lesion glycolysis.

**Fig 9:**
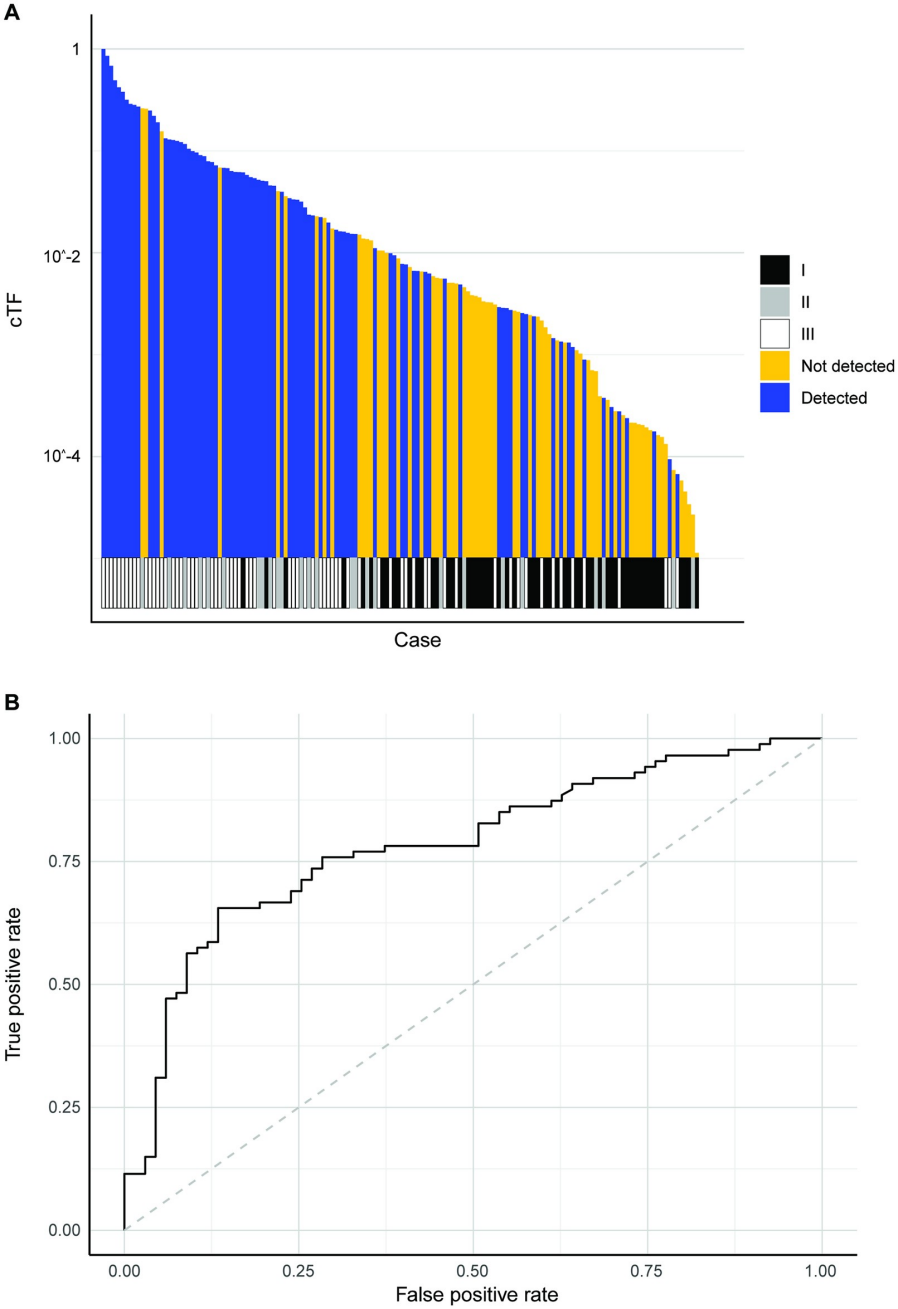
Lung cancer model validation. (A) Detection by TM assay for lung cancer samples sorted by ELG. (B) ROC to predict lung cancer detection by TM assay using ELG. cTF: Circulating tumor fraction, ELG: Excessive lesion glycolysis, ROC: Receiver operating characteristic curve, TM: Targeted methylation.

A linear prediction model was created using ELG (Fig 8). 154 lung cancer cases from the CCGA2 substudy with TM results were available for model validation (13/59 detected in stage I, 17/26 detected in stage II, 57/69 detected in stage III). 3 cases had an imputed size for a non-index lesion, and lymph-node status was derived from clinical stage alone for 2 cases. This model separated detected and undetected cases beyond clinical stage (Fig 9A, Fig B in S1 Text, Wilcoxon rank-sum p-value < 0.001) and can explain lung cancer detection with a TM assay with an AUC of 0.784 (95% CI 0.711–0.857) (Fig 9B).

The corresponding quantitative model estimated that each mm^3^ of additional FDG SUV glycolysis adds 0.81 genome equivalents to the circulation of a cancer patient (p = 0.0004). Assuming a correlation between %Ki-67 positive and FDG PET SUV, the breast and lung cancer models both predict that the total amount of ctDNA increases with tumor volume and mitotic or metabolic tumor activity.

### Colorectal cancer

**Fig 10:**
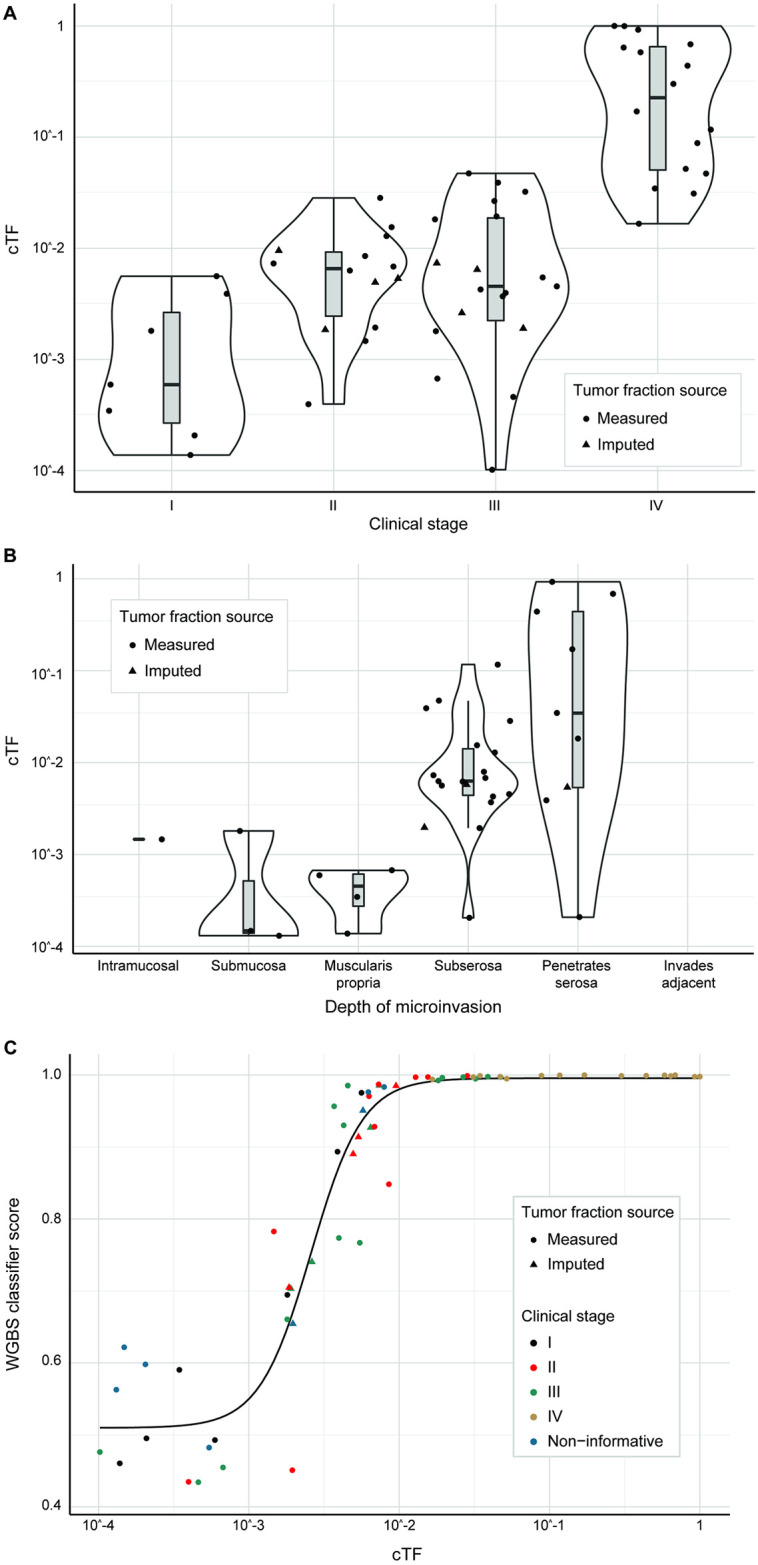
Colorectal cancer cTF distribution by clinical stage, depth of microinvasion, and relation to WGBS classifier score. (A) cTF by clinical stage for colorectal cancer. (B) cTF by depth of microinvasion for colorectal cancer. (C) WGBS classifier score by cTF for colorectal cancer. Samples are colored by clinical stage and samples with imputed cTF are shown as triangles. cTF: Circulating Tumor fraction, WGBS: Whole-genome bisulfite sequencing. Non-informative: Confirmed invasive cancer with insufficient clinical information to determine stage.

Fig 10A shows the distribution of cTF for colorectal cancer cases for clinical stages I-IV, with overlap especially between stages II and III, while Fig 10B shows that depth of microinvasion separates cases with high and low cTF depending on a deep microinvasion beyond the subserosa or a shallow microinvasion below the subserosa. Fig 10C again shows WGBS prototype classifier scores increasing with tumor fraction, the matched sigmoid function, and imputed cTF estimates for this cancer type. 21 colorectal adenocarcinomas (6 stage I, 9 stage II, 6 stage III) had cTF and sufficient clinical information to generate analysis, prediction, and quantitative models for ctDNA. 1 case had cTF imputed from a WGBS classifier score and lymph-node status was derived from clinical stage alone for 1 case.

Table 3 shows the analytical model for colorectal cancer. Only TSA of deeply invading tumors was identified to contribute significantly to cTF, accounting for 75% of explained variability. While not statistically significant, histologic grade and TSA of shallow invading tumors accounted for 12% and 11%, resp.

**Table 3:** Analytical model for colorectal cancer.

Variable	Estimate	p-value	Relative importance
(Intercept)	0.0023	0.6865	NA
Surface area of shallow invading tumors	0.42x10^-6^/mm^2^	0.9280	0.111
Surface area of deep invading tumors	3.52x10^-6^/mm^2^	**0.0422**	0.751
Lymph node status	0.0004	0.9389	0.020
Histologic grade (1 vs. 3/4)	0.0021	0.5011	0.118

NA: not applicable

**Fig 11:**
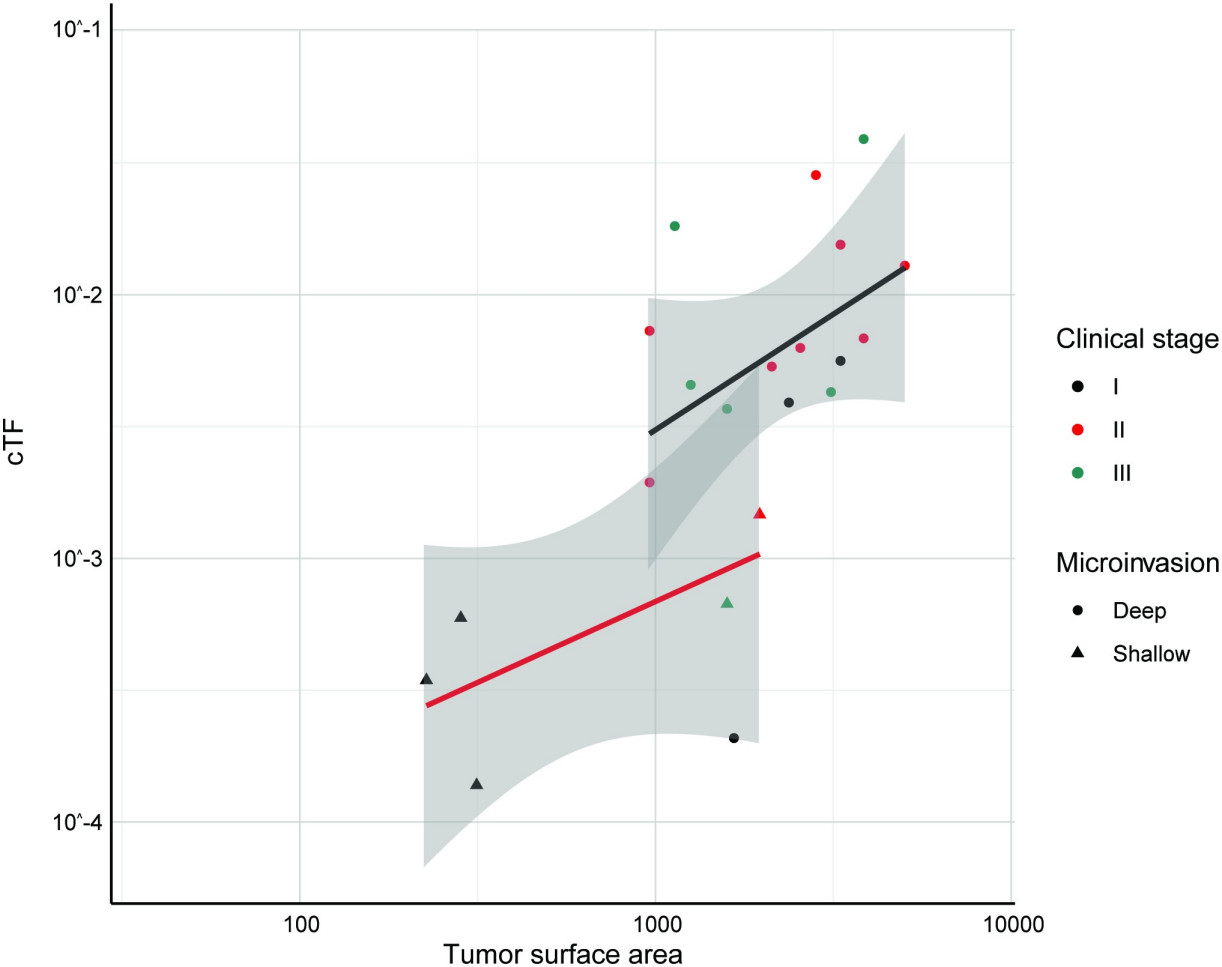
cTF increases with TSA and depth of microinvasion for colorectal cancer. cTF increased with TSA (in mm^2^) and depth of microinvasion for colorectal adenocarcinomas. Dots show cases that invade beyond the subserosa, triangles show cases that do not invade beyond the subserosa. Black, red, and green dots depict samples from stages I, II, and III, respectively. (Model development and validation was limited to clinical stages I-III). Linear fit and 95% confidence intervals are shown in red (shallow microinvasion), black (deep microinvasion), and gray, respectively. cTF: Circulating tumor fraction, TSA: Tumor surface area.

**Fig 12:**
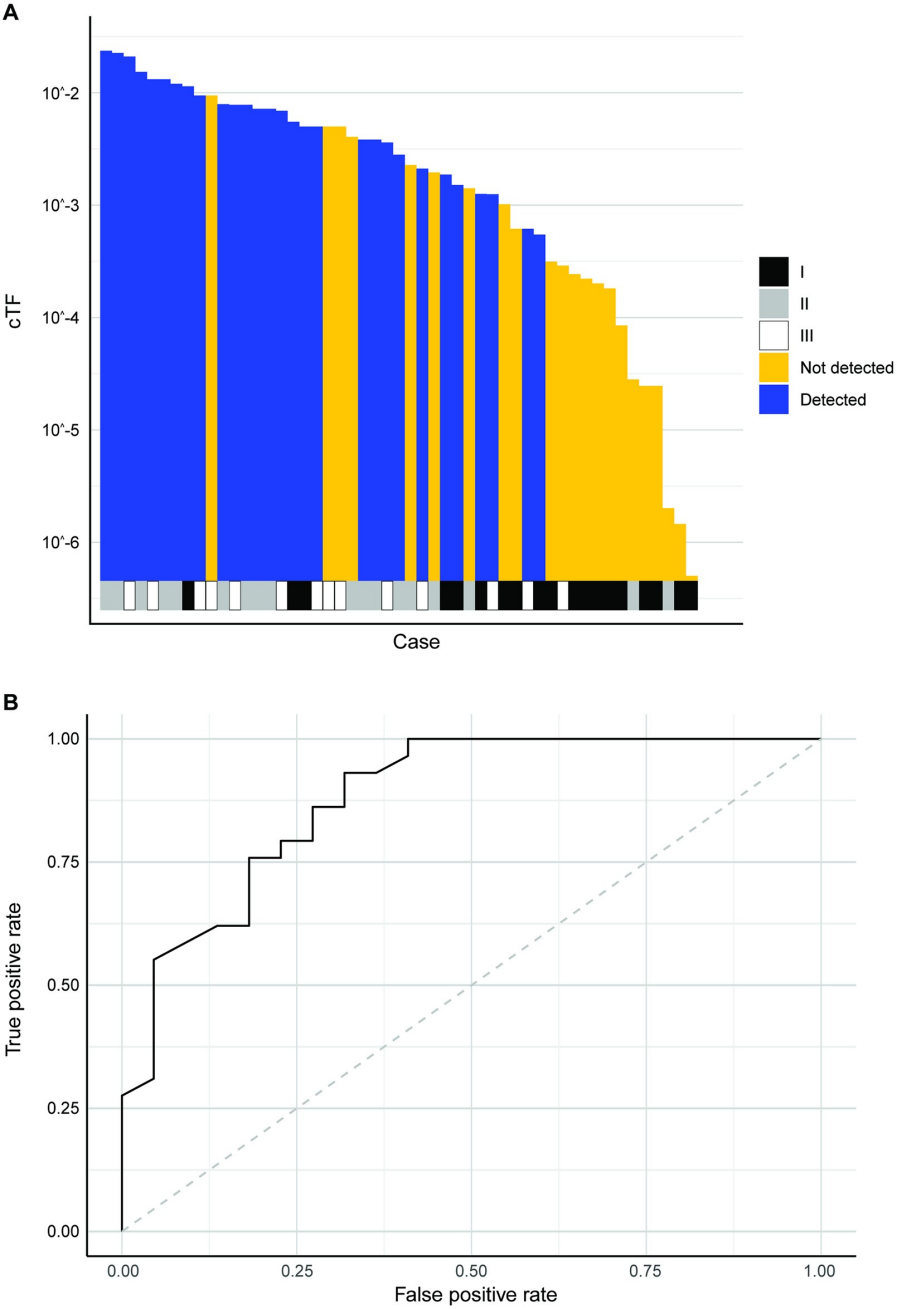
Colorectal cancer model validation. (A) Detection by TM assay for colorectal cancer cases sorted by TSA weighted by depth of microinvasion. Clinical stage is shown at the bottom. (B) ROC to predict colorectal cancer detection by TM assay using weighted TSA. cTF: Circulating tumor fraction, ROC: Receiver operating characteristic curve, TM: Targeted methylation, TSA: Tumor surface area.

Prediction models where cTF increases linearly with TSA were created separately for deep and shallow tumors (Fig 11). 51 colorectal adenocarcinomas from the CCGA2 substudy with TM results were available for model validation (7/19 detected in stage I, 12/18 detected in stage II, 10/14 detected in stage III). 2 cases had an imputed size for a non-index lesion. The predictive model is able to separate detected and undetected cases beyond clinical stage (Fig 12A, Fig C in S1 Text, Wilcoxon rank-sum p-value < 0.001) and explains lung cancer detection with a TM assay with an AUC of 0.881 (95% CI 0.787–0.975) (Fig 12B).

The corresponding quantitative model estimated that each mm^2^ TSA of deeply invading colorectal cancer surface area adds 13.6 genome equivalents to the circulation of a cancer patient (p = 0.0005), while each mm^2^ TSA of a shallowly invading tumor adds only 1.8 genome equivalents (p = 0.014). While again ctDNA increases with the number of tumor cells, in colorectal cancer the trafficking of tumor-derived cfDNA either into the circulation or loss of tumor DNA into the colon lumen are a major correlate of ctDNA.

## Discussion

This study analyzes the nature and pathophysiological correlates of ctDNA. Prediction models were created using tumor-informed cTF measurements and validated on TM assay results from a different participant population. The ability to transfer the models from a training to a separate validation cohort and between different cfDNA assays used for cTF measurements and an application of early cancer detection indicates that the predictive models might represent the cancer biology underlying ctDNA levels.

The identified surrogate biomarkers that determine ctDNA in this post hoc analysis are tumor size, %Ki-67 positive in breast cancer, PET FDG SUV in lung cancer, and depth of microinvasion in colorectal cancer. These are all established prognostic markers of their respective cancer types and assess tumor aggressiveness [51–53]. Here, the biophysical markers TMitV and ELG appeared to capture the essential characteristics of subtypes like HR-negative breast cancer and squamous cell carcinoma of the lung, which have previously been shown to have higher tumor fraction [27–29, 50]. The main link is an apparent correlation between mitotic activity and cell death rates in growing cancers. As explanation, we offer that tumor cells frequently outgrow the tumor mass currently supported by the TME. While the growth of the tumor mass is limited by angiogenesis and scaffolding by growing stroma, tumor cell division in excess of these resource limitations can lead to many forms of division death and release cfDNA into the TME, from where it can get trafficked into the circulation. The models imply a direct link between cell division and cell death in growing tumors. This link is further supported by the possibility of mitotic catastrophes [34], which explain division death even for p53-deficient cells [35]. Mitotically active tumor cells are observed even in hypoxic tumor regions [54].

These pathophysiological mechanisms that link tumor cell growth, tumor cell death, and shedding of ctDNA contradict recently published assumptions of independent tumor growth and cell death rates which have been used to claim that an indolent tumor might result in higher levels of ctDNA than a fast growing tumor [55, 56].

While the identification of clinically reported surrogate biomarkers that correlate with ctDNA levels allows using our models in various applications, the use of surrogate biomarkers is also a study limitation. While the number of tumor cells, the rate of tumor cell death, tumor blood flow and vascular permeability would be physiologically most plausible to quantitatively explain ctDNA, this study aimed to find routinely clinically available markers, identifying tumor size, mitotic or metabolic activity, and depth of invasion as surrogates. We acknowledge that a multi-center observational study like CCGA introduces inter-site variability to clinical data and its completeness for modeling purposes, while a dedicated single-site study might identify additional clinical correlates of ctDNA from stricter controlled quantitative assessments of tumor characteristics.

The CCGA study enrolled participants prior to cancer treatment, and the developed models identify ctDNA correlates in untreated patients. cfDNA-based applications in cancer treatment settings might additionally represent treatment response and resulting tumor cell death [57–59] that are not in scope for the analysis presented here. Furthermore, the developed models are cancer-type-specific and do not yet explain strong variations observed between ctDNA levels for different cancer types [5]. The colorectal cancer model identifies DNA fragment trafficking, (i.e., the transport from the TME into the circulation) as a relevant correlate of ctDNA. Tumor blood flow, perfusion, and vascular permeability are therefore candidates to explain systematic cancer-type-specific variations in ctDNA that have not been assessed in this study. Increased levels of ctDNA for growing tumors can be further explained by tumor perfusion and the dual nature of the vascular epithelial growth factor (VEGF) that is expressed in tumors to drive neovascularization. VEGF has previously been known as vascular permeability factor VPF [60], and increased vascular permeability with increased interstitial filtration flow can contribute to increased trafficking of cfDNA from the tumor mass into the circulation via lymphatic drainage or direct intravasation on the venous side of the vascular perfusion bed. Future work could include relating direct measures of cell death (e.g., using immunohistochemistry assessment of cleaved caspase 3), tumor blood flow, and vascular permeability to ctDNA.

The biophysical models presented here explain levels of ctDNA using only few, physiologically plausible clinical parameters. The identified clinical correlates of ctDNA are at the same time biomarkers of tumor aggressiveness, suggesting that early cancer detection from cfDNA-based applications is determined by tumor growth, i.e., the total number of new tumor cells created in a patient body, instead of tumor size (currently used to characterize image-based screening methods like mammography or low-dose CT [61–63]). This difference in sensitivity either to static tumor size or to tumor growth makes it difficult to compare a cfDNA-based MCED test to existing screening methods, especially as it is meant to complement existing screening methodologies such as mammography. For example, the MCED test used for the validation of the biophysical models in this paper [9, 48] did not detect all early-stage breast cancers that were detected by mammography, but preferably detected more aggressive subtypes [27]. The results in this paper show that cancer detection in cfDNA depends on cTF and TMitV and can be used to explain this behavior of a cfDNA-based MCED test. Together these findings suggest that a cfDNA-based MCED test can complement mammography given that mammography does not detect all fast-growing interval cancers [64, 65] or cancers in women with dense breasts [66, 67]. A recent post-analysis of the second CCGA substudy showed that cancers not detected by a cfDNA-based MCED test had better prognosis than cancers detected by the test, suggesting that the test detected more aggressive cancers [68].

Another systematic difference between imaging- and cfDNA-based cancer early detection is specificity. While consecutive single-cancer screening methods (possibly with comparatively higher sensitivity especially for less aggressive cancers) accumulate false-positive rates [69], one cfDNA MCED test detects all covered cancer types with one single, low false-positive rate. Consequently, it seems more appropriate to characterize a cfDNA-based MCED test not in competition with, but instead as a complement to, imaging-based, single-cancer screening methods. Large studies in a target screening population to assess outcome have recently been announced, and a potential positive impact on patient survival has recently been published using modeled data with the cancer-type and stage-dependent sensitivity of the same test that is used to capture cancer detection in this manuscript [70].

In conclusion, this study supports that tumor fraction plays a pivotal role for cfDNA applications in oncology. It drives the performance of MCED tests, its clinical correlates are indicators of aggressive tumors, and it is ultimately prognostic and identifies potentially lethal cancers [5, 68, 71]. Taken together, these data support that early cancer detection from cfDNA is particularly sensitive to aggressive, fast-growing tumors.

## Supporting information

S1 Text Supplemental methods, results, and data. (DOCX)

S1 Data (ZIP)

## References

[1] Cescon DW, Bratman SV, Chan SM, Siu LL. Circulating tumor DNA and liquid biopsy in oncology. Nat Cancer. 2020;1: 276–290. doi: 10.1038/s43018-020-0043-5 35122035

[2] Corcoran RB. Circulating Tumor DNA: Clinical Monitoring and Early Detection. Annu Rev Cancer Biol. 2019;3: 187–201. doi: 10.1146/annurev-cancerbio-030518-055719

[3] Bettegowda C, Sausen M, Leary RJ, Kinde I, Wang Y, Agrawal N, et al. Detection of circulating tumor DNA in early- and late-stage human malignancies. Sci Transl Med. 2014;6: 224ra24. doi: 10.1126/scitranslmed.3007094 24553385 PMC4017867

[4] Diehl F, Li M, Dressman D, He Y, Shen D, Szabo S, et al. Detection and quantification of mutations in the plasma of patients with colorectal tumors. Proc Natl Acad Sci. 2005;102: 16368–16373. doi: 10.1073/pnas.0507904102 16258065 PMC1283450

[5] Venn O, Hubbell E, Sakarya O, Chang C, Halks-Miller M, Steffen K, et al. Tumor shedding into cell-free DNA (cfDNA) is associated with high-mortality cancers [poster]. 31st Annual Meeting on The Biology of Genomes. Cold Spring Harbor, NY: Cold Spring Harbor Laboratory; 2019. https://meetings.cshl.edu/abstracts.aspx?meet=GENOME&year=19

[6] Cohen JD, Li L, Wang Y, Thoburn C, Afsari B, Danilova L, et al. Detection and localization of surgically resectable cancers with a multi-analyte blood test. Science. 2018;359: 926–930. doi: 10.1126/science.aar3247 29348365 PMC6080308

[7] Shen SY, Singhania R, Fehringer G, Chakravarthy A, Roehrl MHA, Chadwick D, et al. Sensitive tumour detection and classification using plasma cell-free DNA methylomes. Nature. 2018;563: 579–583. doi: 10.1038/s41586-018-0703-0 30429608

[8] Cristiano S, Leal A, Phallen J, Fiksel J, Adleff V, Bruhm DC, et al. Genome-wide cell-free DNA fragmentation in patients with cancer. Nature. 2019;570: 385–389. doi: 10.1038/s41586-019-1272-6 31142840 PMC6774252

[9] Liu MC, Oxnard GR, Klein EA, Swanton C, Seiden MV, Cummings SR, et al. Sensitive and specific multi-cancer detection and localization using methylation signatures in cell-free DNA. Ann Oncol. 2020;31: 745–759. doi: 10.1016/j.annonc.2020.02.011 33506766 PMC8274402

[10] Zviran A, Schulman RC, Shah M, Hill STK, Deochand S, Khamnei CC, et al. Genome-wide cell-free DNA mutational integration enables ultra-sensitive cancer monitoring. Nat Med. 2020;26: 1114–1124. doi: 10.1038/s41591-020-0915-3 32483360 PMC8108131

[11] Smith JT, Balar A, Lakhani DA, Kluwe C, Zhao Z, Kopparapu P, et al. Circulating tumor DNA as a potential biomarker of radiographic tumor burden in small cell lung cancer. Cancer Res. 2020;80 (16 supplement). Abstract 715. doi: 10.1158/1538-7445.AM2020-715

[12] Reinert T, Henriksen TV, Christensen E, Sharma S, Salari R, Sethi H, et al. Analysis of Plasma Cell-Free DNA by Ultradeep Sequencing in Patients With Stages I to III Colorectal Cancer. JAMA Oncol. 2019;5: 1124–1131. doi: 10.1001/jamaoncol.2019.0528 31070691 PMC6512280

[13] McDonald BR, Contente-Cuomo T, Sammut S-J, Odenheimer-Bergman A, Ernst B, Perdigones N, et al. Personalized circulating tumor DNA analysis to detect residual disease after neoadjuvant therapy in breast cancer. Sci Transl Med. 2019;11. doi: 10.1126/scitranslmed.aax7392 31391323 PMC7236617

[14] Heitzer E, Auinger L, Speicher MR. Cell-Free DNA and Apoptosis: How Dead Cells Inform About the Living. Trends Mol Med. 2020;26: 519–528. doi: 10.1016/j.molmed.2020.01.012 32359482

[15] Bronkhorst AJ, Wentzel JF, Aucamp J, van Dyk E, du Plessis L, Pretorius PJ. Characterization of the cell-free DNA released by cultured cancer cells. Biochim Biophys Acta. 2016;1863: 157–165. doi: 10.1016/j.bbamcr.2015.10.022 26529550

[16] Elshimali YI, Khaddour H, Sarkissyan M, Wu Y, Vadgama JV. The Clinical Utilization of Circulating Cell Free DNA (CCFDNA) in Blood of Cancer Patients. Int J Mol Sci. 2013;14: 18925–18958. doi: 10.3390/ijms140918925 24065096 PMC3794814

[17] Diaz LA, Bardelli A. Liquid Biopsies: Genotyping Circulating Tumor DNA. J Clin Oncol. 2014;32: 579–586. doi: 10.1200/JCO.2012.45.2011 24449238 PMC4820760

[18] Aalipour A, Chuang H-Y, Murty S, D’Souza AL, Park S, Gulati GS, et al. Engineered immune cells as highly sensitive cancer diagnostics. Nat Biotechnol. 2019;37: 531–539. doi: 10.1038/s41587-019-0064-8 30886438 PMC7295609

[19] Han DSC, Ni M, Chan RWY, Chan VWH, Lui KO, Chiu RWK, et al. The Biology of Cell-free DNA Fragmentation and the Roles of DNASE1, DNASE1L3, and DFFB. Am J Hum Genet. 2020;106: 202–214. doi: 10.1016/j.ajhg.2020.01.008 32004449 PMC7010979

[20] Schwarzenbach H, Hoon DSB, Pantel K. Cell-free nucleic acids as biomarkers in cancer patients. Nat Rev Cancer. 2011;11: 426–437. doi: 10.1038/nrc3066 21562580

[21] Jahr S, Hentze H, Englisch S, Hardt D, Fackelmayer FO, Hesch RD, et al. DNA fragments in the blood plasma of cancer patients: quantitations and evidence for their origin from apoptotic and necrotic cells. Cancer Res. 2001;61: 1659–1665. 11245480

[22] Rew DA, Wilson GD. Cell production rates in human tissues and tumours and their significance. Part 1: an introduction to the techniques of measurement and their limitations. Eur J Surg Oncol. 2000;26: 227–238. doi: 10.1053/ejso.1999.0781 10753534

[23] Kustanovich A, Schwartz R, Peretz T, Grinshpun A. Life and death of circulating cell-free DNA. Cancer Biol Ther. 2019;20: 1057–1067. doi: 10.1080/15384047.2019.1598759 30990132 PMC6606043

[24] Srivastava S, Koay EJ, Borowsky AD, De Marzo AM, Ghosh S, Wagner PD, et al. Cancer overdiagnosis: a biological challenge and clinical dilemma. Nat Rev Cancer. 2019;19: 349–358. doi: 10.1038/s41568-019-0142-8 31024081 PMC8819710

[25] Siegel RL, Miller KD, Jemal A. Cancer statistics, 2020. CA Cancer J Clin. 2020;70: 7–30. doi: 10.3322/caac.21590 31912902

[26] Klein EA, Hubbell E, Maddala T, Aravanis A, Beausang JF, Filippova D, et al. Development of a comprehensive cell-free DNA (cfDNA) assay for early detection of multiple tumor types: The Circulating Cell-free Genome Atlas (CCGA) study. J Clin Oncol. 2018;36: 12021–12021. doi: 10.1200/JCO.2018.36.15_suppl.12021

[27] Liu MC, Carter JM, Visscher DW, Kopp K, Shaknovich R, Chen X, et al. Blood-based cancer detection in plasma cell-free DNA (cfDNA): evaluating clinical and pathologic tumor characteristics in participants with breast cancer. Cancer Res. 2020;80(4 supplement): P5-01-01-P5-01-01. doi: 10.1158/1538-7445.SABCS19-P5-01-01

[28] Abbosh C, Birkbak NJ, Wilson GA, Jamal-Hanjani M, Constantin T, Salari R, et al. Phylogenetic ctDNA analysis depicts early-stage lung cancer evolution. Nature. 2017;545: 446–451. doi: 10.1038/nature22364 28445469 PMC5812436

[29] Chabon JJ, Hamilton EG, Kurtz DM, Esfahani MS, Moding EJ, Stehr H, et al. Integrating genomic features for non-invasive early lung cancer detection. Nature. 2020;580: 245–251. doi: 10.1038/s41586-020-2140-0 32269342 PMC8230734

[30] Bredno J, Lipson J, Venn O, Gross S, Fields AP, Beausang JF, et al. Tumor area and microscopic extent of invasion to determine circulating tumor DNA fraction in plasma and detectability of colorectal cancer (CRC). J Clin Oncol. 2020;38: 243–243. doi: 10.1200/JCO.2020.38.4_suppl.243

[31] Nadler SB, Hidalgo JH, Bloch T. Prediction of blood volume in normal human adults. Surgery. 1962;51: 224–232. 21936146

[32] Stover DG, Parsons HA, Ha G, Freeman SS, Barry WT, Guo H, et al. Association of cell-free DNA tumor fraction and somatic copy number alterations with survival in metastatic triple-negative breast cancer. J Clin Oncol. 2018;36: 543–553. doi: 10.1200/JCO.2017.76.0033 29298117 PMC5815405

[33] Amin MB, Greene FL, Edge SB, Compton CC, Gershenwald JE, Brookland RK, et al. The Eighth Edition AJCC Cancer Staging Manual: continuing to build a bridge from a population-based to a more “personalized” approach to cancer staging. CA Cancer J Clin. 2017;67: 93–99. doi: 10.3322/caac.21388 28094848

[34] Castedo M, Perfettini J-L, Roumier T, Andreau K, Medema R, Kroemer G. Cell death by mitotic catastrophe: a molecular definition. Oncogene. 2004;23: 2825–2837. doi: 10.1038/sj.onc.1207528 15077146

[35] Vakifahmetoglu H, Olsson M, Zhivotovsky B. Death through a tragedy: mitotic catastrophe. Cell Death Differ. 2008;15: 1153–1162. doi: 10.1038/cdd.2008.47 18404154

[36] van der Pol Y, Mouliere F. Toward the Early Detection of Cancer by Decoding the Epigenetic and Environmental Fingerprints of Cell-Free DNA. Cancer Cell. 2019;36: 350–368. doi: 10.1016/j.ccell.2019.09.003 31614115

[37] Liu Z, Jiao D. Necroptosis, tumor necrosis and tumorigenesis. Cell Stress. 2020;4: 1–8. doi: 10.15698/cst2020.01.208 31922095 PMC6946014

[38] Lindeman RH, Merenda PF, Gold RZ. Introduction to Bivariate and Multivariate Analysis. Glenview, IL: Scott, Foresman and Company; 1980.

[39] Chevan A, Sutherland M. Hierarchical Partitioning. Am Stat. 1991;45: 90–96. doi: 10.1080/00031305.1991.10475776

[40] Deniz F, Dilek K, Hande M, Umit UM, Handan K. Ki-67 and caspase expression in breast carcinoma: does variance in locational sampling exist? Int J Clin Exp Pathol. 2015;8: 11305–11313. 26617854 PMC4637670

[41] Duhaylongsod FG, Lowe VJ, Patz EF, Vaughn AL, Coleman RE, Wolfe WG. Lung tumor growth correlates with glucose metabolism measured by fluoride-18 fluorodeoxyglucose positron emission tomography. Ann Thorac Surg. 1995;60: 1348–1352. doi: 10.1016/0003-4975(95)00754-9 8526625

[42] Tann M, Sandrasegaran K, Winer-Muram HT, Jennings SG, Welling ME, Fletcher JW. Can FDG-PET be used to predict growth of stage I lung cancer? Clin Radiol. 2008;63: 856–863. doi: 10.1016/j.crad.2008.01.012 18625349

[43] Cochet A, Pigeonnat S, Khoury B, Vrigneaud J-M, Touzery C, Berriolo-Riedinger A, et al. Evaluation of Breast Tumor Blood Flow with Dynamic First-Pass 18F-FDG PET/CT: Comparison with Angiogenesis Markers and Prognostic Factors. J Nucl Med. 2012;53: 512–520. doi: 10.2967/jnumed.111.096834 22343501

[44] Sood A, Miller AM, Brogi E, Sui Y, Armenia J, McDonough E, et al. Multiplexed immunofluorescence delineates proteomic cancer cell states associated with metabolism. JCI Insight. 2016;1. doi: 10.1172/jci.insight.87030 27182557 PMC4863708

[45] Vesselle H, Schmidt RA, Pugsley JM, Li M, Kohlmyer SG, Vallières E, et al. Lung Cancer Proliferation Correlates with [F-18]Fluorodeoxyglucose Uptake by Positron Emission Tomography. Clin Cancer Res. 2000;6: 3837–3844. 11051227

[46] Nygaard AD, Holdgaard PC, Spindler K-LG, Pallisgaard N, Jakobsen A. The correlation between cell-free DNA and tumour burden was estimated by PET/CT in patients with advanced NSCLC. Br J Cancer. 2014;110: 363–368. doi: 10.1038/bjc.2013.705 24231948 PMC3899755

[47] Moss J, Magenheim J, Neiman D, Zemmour H, Loyfer N, Korach A, et al. Comprehensive human cell-type methylation atlas reveals origins of circulating cell-free DNA in health and disease. Nat Commun. 2018;9: 5068. doi: 10.1038/s41467-018-07466-6 30498206 PMC6265251

[48] Klein EA, Richards D, Cohn A, Tummala M, Lapham R, Cosgrove D, et al. Clinical validation of a targeted methylation-based multi-cancer early detection test using an independent validation set. Ann Oncol. 2021; doi: 10.1016/j.annonc.2021.05.806 34176681

[49] Beer TM, McDonnell CH, Nadauld L, Liu MC, Klein EA, Reid RL, et al. Interim results of PATHFINDER, a clinical use study using a methylation-based multi-cancer early detection test. J Clin Oncol. 2021;39: 3010–3010. doi: 10.1200/JCO.2021.39.15_suppl.3010

[50] Jiang J, Adams H-P, Yao L, Yaung S, Lal P, Balasubramanyam A, et al. Concordance of Genomic Alterations by Next-Generation Sequencing in Tumor Tissue versus Cell-Free DNA in Stage I–IV Non–Small Cell Lung Cancer. J Mol Diagn. 2020;22: 228–235. doi: 10.1016/j.jmoldx.2019.10.013 31837429

[51] Berghmans T, Dusart M, Paesmans M, Hossein-Foucher C, Buvat I, Castaigne C, et al. Primary tumor standardized uptake value (SUVmax) measured on fluorodeoxyglucose positron emission tomography (FDG-PET) is of prognostic value for survival in non-small cell lung cancer (NSCLC): a systematic review and meta-analysis (MA) by the European Lung Cancer Working Party for the IASLC Lung Cancer Staging Project. J Thorac Oncol Off Publ Int Assoc Study Lung Cancer. 2008;3: 6–12. doi: 10.1097/JTO.0b013e31815e6d6b 18166834

[52] Inwald EC, Klinkhammer-Schalke M, Hofstädter F, Zeman F, Koller M, Gerstenhauer M, et al. Ki-67 is a prognostic parameter in breast cancer patients: results of a large population-based cohort of a cancer registry. Breast Cancer Res Treat. 2013;139: 539–552. doi: 10.1007/s10549-013-2560-8 23674192 PMC3669503

[53] Li J, Guo B-C, Sun L-R, Wang J-W, Fu X-H, Zhang S-Z, et al. TNM staging of colorectal cancer should be reconsidered by T stage weighting. World J Gastroenterol. 2014;20: 5104–5112. doi: 10.3748/wjg.v20.i17.5104 24803826 PMC4009548

[54] Vega Harring S, Canamero M, Korski K, Marchal G, Grimm O, Ferreira C, et al. Unraveling tumor metabolism with in silico IHC multiplexing supported by automated imaging analysis. Mol Cancer Res. 2016;14: B47–B47. doi: 10.1158/1557-3125.METCA15-B47

[55] Avanzini S, Kurtz DM, Chabon JJ, Moding EJ, Hori SS, Gambhir SS, et al. A mathematical model of ctDNA shedding predicts tumor detection size. Sci Adv. 2020;6: eabc4308. doi: 10.1126/sciadv.abc4308 33310847 PMC7732186

[56] Pashayan N, Pharoah PDP. The challenge of early detection in cancer. Science. 2020;368: 589–590. doi: 10.1126/science.aaz2078 32381710

[57] Deligezer U, Eralp Y, Akisik EE, Akisik EZ, Saip P, Topuz E, et al. Size distribution of circulating cell-free DNA in sera of breast cancer patients in the course of adjuvant chemotherapy. Clin Chem Lab Med. 2008;46: 311–317. doi: 10.1515/CCLM.2008.080 18254709

[58] Kwee S, Song M-A, Cheng I, Loo L, Tiirikainen M. Measurement of Circulating Cell-Free DNA in Relation to 18F-Fluorocholine PET/CT Imaging in Chemotherapy-Treated Advanced Prostate Cancer. Clin Transl Sci. 2012;5: 65–70. doi: 10.1111/j.1752-8062.2011.00375.x 22376260 PMC3500883

[59] Sterzik A, Paprottka PM, Zengel P, Hirner H, Roßpunt S, Eschbach R, et al. DCE-MRI biomarkers for monitoring an anti-angiogenic triple combination therapy in experimental hypopharynx carcinoma xenografts with immunohistochemical validation. Acta Radiol. 2015;56: 294–303. doi: 10.1177/0284185114527444 24609871

[60] Dvorak HF. Vascular Permeability Factor/Vascular Endothelial Growth Factor: A Critical Cytokine in Tumor Angiogenesis and a Potential Target for Diagnosis and Therapy. J Clin Oncol. 2002;20: 4368–4380. doi: 10.1200/JCO.2002.10.088 12409337

[61] Han D, Heuvelmans MA, Oudkerk M. Volume versus diameter assessment of small pulmonary nodules in CT lung cancer screening. Transl Lung Cancer Res. 2017;6: 52–61–61. doi: 10.21037/tlcr.2017.01.05 28331824 PMC5344834

[62] Welch HG, Prorok PC, O’Malley AJ, Kramer BS. Breast-Cancer Tumor Size, Overdiagnosis, and Mammography Screening Effectiveness. N Engl J Med. 2016;375: 1438–1447. doi: 10.1056/NEJMoa1600249 27732805

[63] Cortadellas T, Argacha P, Acosta J, Rabasa J, Peiró R, Gomez M, et al. Estimation of tumor size in breast cancer comparing clinical examination, mammography, ultrasound and MRI—correlation with the pathological analysis of the surgical specimen. Gland Surg. 2017;6: 330–335. doi: 10.21037/gs.2017.03.09 28861372 PMC5566672

[64] Houssami N, Hunter K. The epidemiology, radiology and biological characteristics of interval breast cancers in population mammography screening. Npj Breast Cancer. 2017;3: 1–13. 28649652 10.1038/s41523-017-0014-x PMC5460204

[65] Niraula S, Biswanger N, Hu P, Lambert P, Decker K. Incidence, Characteristics, and Outcomes of Interval Breast Cancers Compared With Screening-Detected Breast Cancers. JAMA Netw Open. 2020;3: e2018179–e2018179. doi: 10.1001/jamanetworkopen.2020.18179 32975573 PMC7519419

[66] Burnside ES, Warren LM, Myles J, Wilkinson LS, Wallis MG, Patel M, et al. Quantitative breast density analysis to predict interval and node-positive cancers in pursuit of improved screening protocols: a case–control study. Br J Cancer. 2021; 1–9. doi: 10.1038/s41416-021-01466-y 34168297 PMC8438060

[67] Bakker MF, de Lange SV, Pijnappel RM, Mann RM, Peeters PHM, Monninkhof EM, et al. Supplemental MRI screening for women with extremely dense breast tissue. N Engl J Med. 2019;381: 2091–2102. doi: 10.1056/NEJMoa1903986 31774954

[68] Chen X, Dong Z, Hubbell E, Kurtzman KN, Oxnard GR, Venn O, et al. Prognostic Significance of Blood-Based Multi-cancer Detection in Plasma Cell-Free DNA. Clin Cancer Res. 2021 [cited 24 Jun 2021]. Epub 4 Jun 2021. doi: 10.1158/1078-0432.CCR-21-0417 34088722 PMC9401481

[69] Clarke CA, Hubbell E, Ofman JJ. Multi-cancer early detection: A new paradigm for reducing cancer-specific and all-cause mortality. Cancer Cell. 2021;39: 447–448. doi: 10.1016/j.ccell.2021.02.004 33606995

[70] Hubbell E, Clarke CA, Aravanis AM, Berg CD. Modeled Reductions in Late-stage Cancer with a Multi-Cancer Early Detection Test. Cancer Epidemiol Biomarkers Prev. 2021;30: 460–468. doi: 10.1158/1055-9965.EPI-20-1134 33328254

[71] Oxnard GR, Chen X, Fung ET, Ma T, Lipson J, Hubbell E, et al. Prognostic significance of blood-based cancer detection in plasma cell-free DNA (cfDNA): evaluating risk of overdiagnosis. J Clin Oncol. 2019;37: Abstract 1545. doi: 10.1200/JCO.2019.37.15_suppl.1545

