# Supplementary material for: Clinical correlates of circulating cell-free DNA tumor fraction
Source: PLoS One. 2021 Aug 25;16(8):e0256436. doi: 10.1371/journal.pone.0256436 (PMC8386888; doi:10.1371/journal.pone.0256436)
Supplement: S1 Text — (DOCX) [file pone.0256436.s001.docx]

### **Supporting Information**

### **Supplemental methods**

To test if either tumor size or tumor mitotic or metabolic activity, which represent tumor growth are the main correlate of circulating tumor DNA (ctDNA), additional linear analysis models were created offering both, total volume of primary tumor lesions and tumor mitotic volume (TMitV) and excessive lesion glycolysis (ELG), respectively as covariates.

To confirm if the analysis models can explain circulating tumor fraction (cTF) and the cancer detection of the targeted methylation (TM) assay beyond clinical stage, receiver operating characteristic (ROC) curves to predict cancer detection were created for each tumor separately for clinical stage I, II, III.

### **Supplemental results**

A competing unconstrained analysis model for breast cancer built from TMitV, total volume of primary tumor, presence of tumor-involved lymph nodes, histologic grade, and histologic cancer type continued to identify TMitV, but not total volume as significant correlate of cTF (Table A). In this competing model, TMitV accounted for 35% of explained variability while primary volume would have a negative, not significant contribution.

**Table A. Competing Model Testing the Influence of Primary Tumor Volume and TMitV.**

| **Variable** | **Estimate** | **p-value** | **Relative Importance** |
| --- | --- | --- | --- |
| (Intercept) | 0.0050 | 0.7373 | NA |
| TMitV | 8.08x10^–7^/mm^3^ | **0.0134** | 0.350 |
| Primary volume | –2.71x10^–7^/mm^3^ | 0.0894 | 0.192 |
| Lymph node status | 0.0169 | 0.0623 | 0.174 |
| Hormone receptor status | –0.0044 | 0.4509 | 0.104 |
| Histologic grade | 0.0071 | 0.2423 | 0.158 |
| Invasive lobular carcinoma | –0.0004 | 0.9822 | 0.009 |
| Invasive ductal carcinoma | 0.0035 | 0.8127 | 0.013 |

NA, not applicable, TMitV: tumor mitotic volume

For breast cancers, TMitV was able to predict detection of stage II cancers with an ROC of 0.746 (95% CI 0.627 - 0.865) and separated detected (28/67) from non-detected cases (Wilcoxon rank-sum p-value = 0.0003). No meaningful results were obtained for stages I and III which have respective very low and very high sensitivity (1/65 detected and 13/14 detected) that impede a meaningful ROC computation (Fig A).

**Fig A. Breast Cancer Model Validation by Clinical Stage.** ROC to predict breast cancer detection by TM assay using TMitV with an additional breakdown limiting samples to stage I (red), stage II (green) and stage III (beige), respectively. TM: Targeted methylation, ROC: Receiver operating characteristic curve. TMitV: Tumor mitotic volume.

| 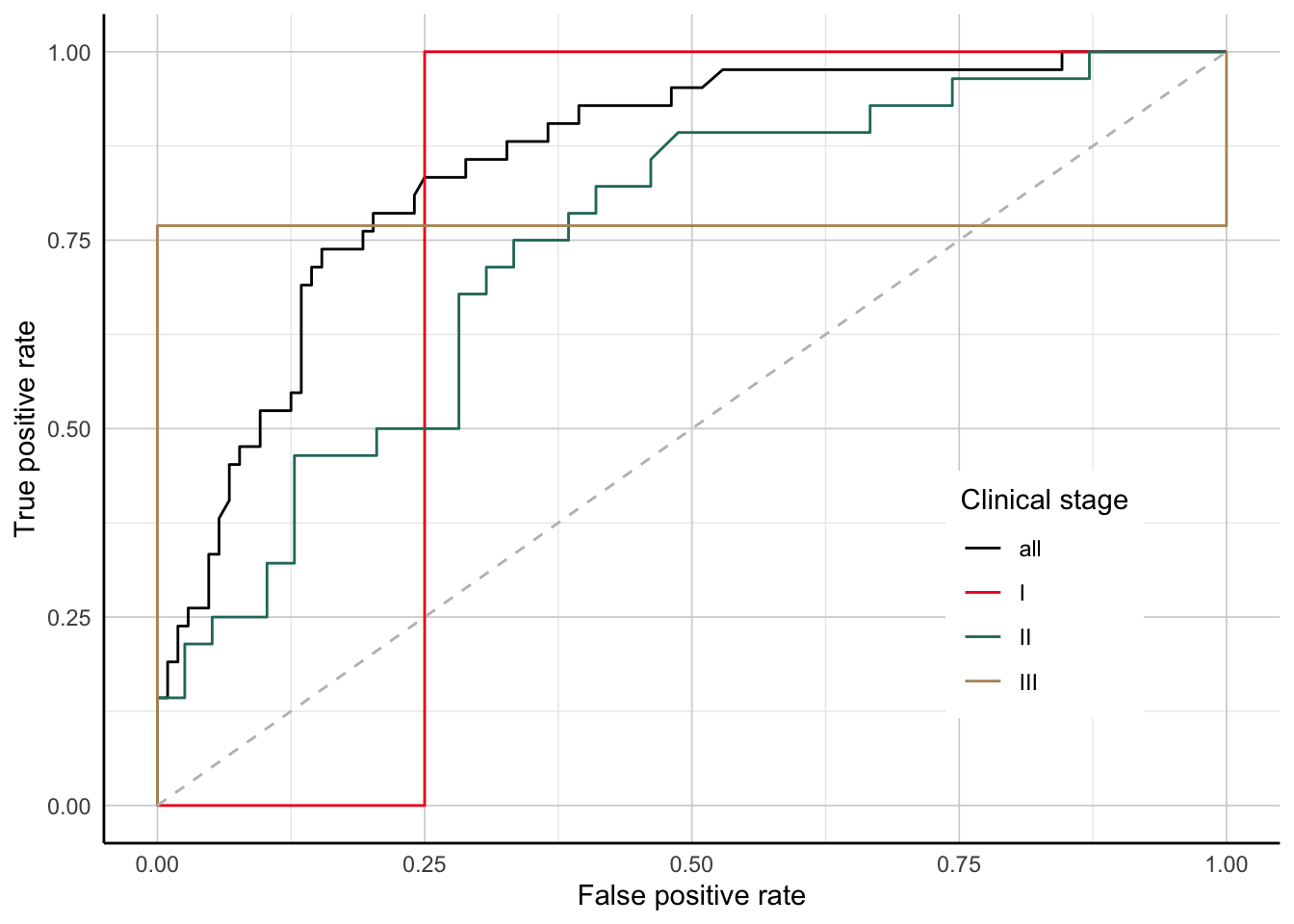 |
| --- |

A competing unconstrained analysis model for lung cancer built from ELG, total volume of primary tumor, presence of tumor-involved lymph nodes, histologic grade, and histologic cancer type identified both, ELG and primary volume as strong contributors to explained variation (45% and 41%, respectively), but the close relation between these tumor characteristics did not identify a single covariate as statistically significant (p-values 0.125 and 0.640, respectively, Table B). In a linear multivariable model created with primary tumor volume instead of ELG, tumor volume accounted for 64% of all explained variability, while ELG alone accounted for 81% of explained variability.

**Table B. Competing Model Testing the Influence of Primary Tumor Volume and ELG.**

| **Variable** | **Estimate** | **p-value** | **Relative Importance** |
| --- | --- | --- | --- |
| (Intercept) | 0.0138 | 0.8848 | NA |
| ELG | 48.5x10^-9^ / mm^3^ | 0.1249 | 0.449 |
| Primary volume | 279x10^-9^ / mm^3^ | 0.6398 | 0.410 |
| Lymph node status | 0.0024 | 0.9660 | 0.022 |
| Histologic grade | 0.0386 | 0.4602 | 0.099 |
| Adenocarcinoma | -0.0086 | 0.9274 | 0.0033 |
| Squamous cell carcinoma | -0.0578 | 0.5619 | 0.010 |
| Small cell carcinoma | 0.0490 | 0.6884 | 0.0075 |

ELG: Excessive lesion glycolysis, NA, not applicable.

For lung cancers, ELG explained detection by the TM assay separately by stage with an area under the curve (AUC) of 0.640, 0.719, and 0.662, for stages I, II, and III, respectively (Fig B). ELG separated detected from non-detected cases separately at stages II (17/26 detected, Wilcoxon rank-sum p-value 0.0375) and III (57/69 detected, Wilcoxon rank-sum p-value = 0.04), respectively, while the separation was not significant for stage I (13/59 detected, Wilcoxon rank-sum p = 0.0645).

**Fig B. Lung Cancer Model Validation by Clinical Stage.** ROC to predict lung cancer detection by TM assay using ELG with an additional breakdown limiting samples to stage I (red), stage II (green) and stage III (beige), respectively. TM: Targeted methylation, ELG: Excessive lesion glycolysis, ROC: Receiver operating characteristic curve.

| 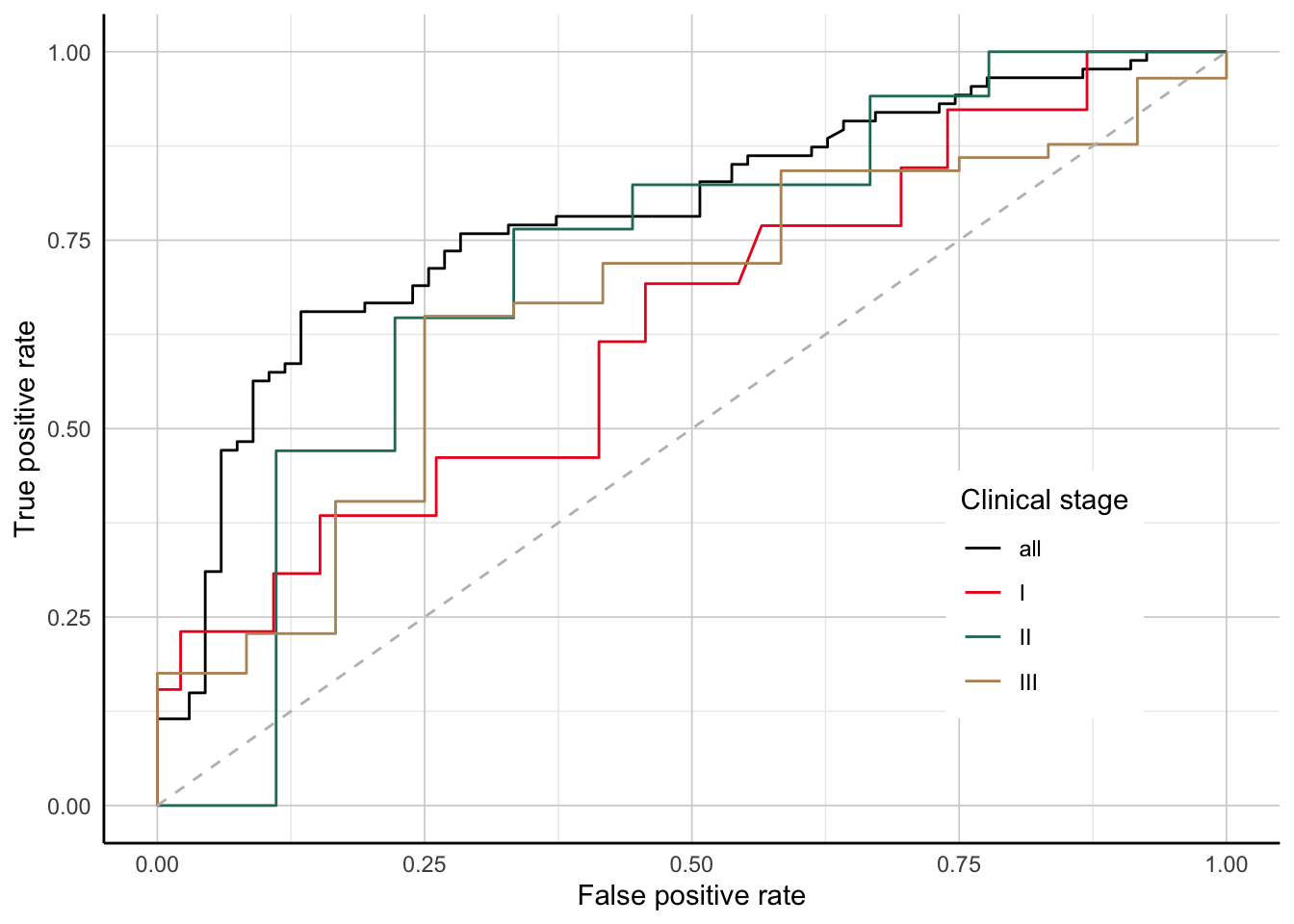 |
| --- |

For colorectal adenocarcinoma, tumor surface area (TSA) and depth of microinvasion explained detection by the TM assay separately by stage with an AUC of 0.976, 0.958, and 0.588, for stages I, II, and III, respectively (Fig C). The model separated detected from non-detected cases at stages I (7/19 detected, Wilcoxon rank-sum p-value 0.0004) and II (12/18 detected, Wilcoxon rank-sum p = 0.0012), respectively. The lower AUC and non-significant p-value of the Wilcoxon test (p = 0.335, 10/14 detected) for stage III cancers might be explained by a low number of cases and some variability of ctDNA from tumor-involved lymph nodes.

**Fig C. Colorectal Cancer Model Validation by Clinical Stage.** ROC to predict colorectal cancer detection by TM assay using tumor area weighted by depth of microinvasion with an additional breakdown limiting samples to stage I (red), stage II (green) and stage III (beige), respectively. TM: Targeted methylation, ROC: Receiver operating characteristic curve.

| 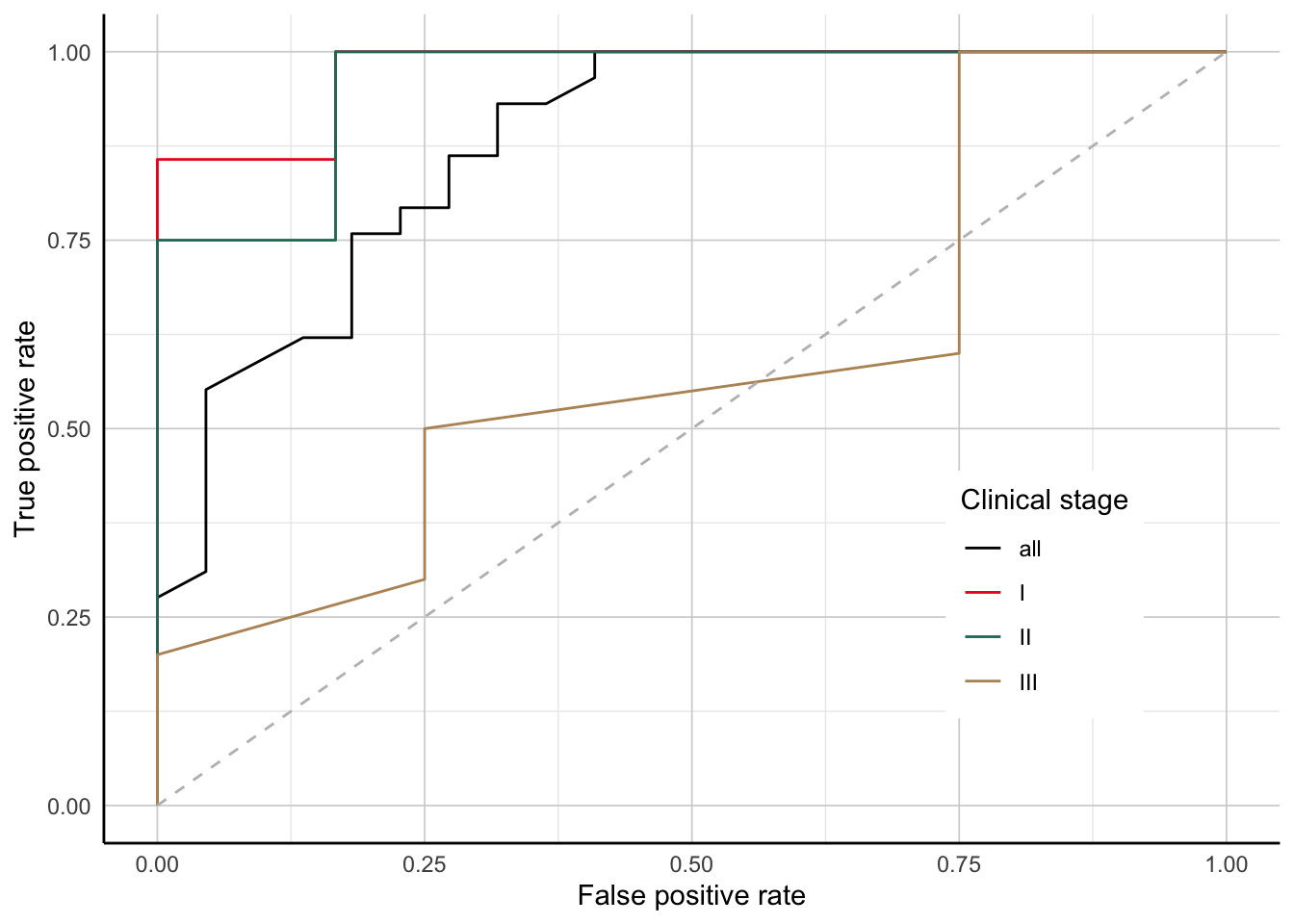 |
| --- |

### **Supplemental data**

The data used in the presented analyses are provided in tables separate for breast, lung, and colorectal cancers and for participants used in training and validation. The tables have one row per participant and data in the following columns:

| participant_id | Study-specific participant ID assigned at enrollment |
| --- | --- |
| sex | Participant sex, "Female" or "Male" |
| age | Participant age at enrollment |
| weight_kg | Participant weight in kg |
| height_m | Participant height in m |
| clinical_stage | Clinical stage reported by enrolling site |
| t_stage | Clinical T-stage reported by enrolling site |
| n_stage | Clinical N-stage reported by enrolling site |
| m_stage | Clinical M-stage reported by enrolling site |
| ln_involved | Number of tumor-involved lymph nodes from path report. Results from multiple primary lesions are separated by comma. |
| laterality | (breast cancer only) "bilateral" or "unilateral" |
| n_primary_lesions | Derived number of primary lesion foci. Information is accumulated for bilateral disease, and one primary focus per side is assumed when information on lesion focality is not reported by enrolling site or path report. |
| size_1 | Maximum extent of largest primary tumor focus in mm |
| size_2, size_3, size_4 | Maximum extent of 2nd and 3rd, 4th largest primary tumor focus, if reported. |
| histologic_grade | Grade from path report |
| histologic_type | Histologic type. For breast cancers, this is "IDC" for invasive ductal carcinoma, "ILC" for invasive lobular carcinoma, or "Other" for breast cancers that are neither ILC or IDC. For lung cancers, this is "ADC" for adenocarcinomas, "SCC" for squamous cell carcinomas, "SCLC" for small cell lung cancer and "Other" for all other cancer types including NSCLC NOS (non-small cell lung cancer not otherwise specified). This column is not provided for colorectal cancers as only adenocarcinomas were included in that analysis |
| ki67_pos | (breast cancer only) %positive Ki-67 reported by enrolling site or result of additional IHC (ImmunoHistoChemistry) testing |
| hr_status | (breast cancer only) Hormone receptor status. "Negative" if at least one of possibly multiple lesions is hormone-receptor negative. A lesion is hormone-receptor negative if it is negative for estrogen and progesterone receptor overexpression. |
| fdg_suv | (lung cancer only) FDG PET ([fluorodeoxyglucose positron emission tomography](https://www.zotero.org/google-docs/?CkLIGC)) Standardized uptake value of the index lesion |
| microinvasion | (colorectal cancer only) Depth of microscopic microinvasion from path report. |
| ctf | (training data only) Circulating tumor fraction |
| ctf_source | (training data only) Either "measured" or "imputed". cTF was measured as presented in Venn, et al. [[5]](https://www.zotero.org/google-docs/?ANuki5). Imputed data was determined from a whole-genome bisulfite sequencing (WGBS) classifier score (Figures 4B, 7B, 10C, resp.) |
| wgbs_classifier_score | (training data only) Cancer score from the classifier trained from abnormally methylated fragments in a WGBS assay [[26]](https://www.zotero.org/google-docs/?iZ2W6b) |
| tm_classifier_result | (validation data only) Cancer signal detection from targeted methylation assay and classifier [[9]](https://www.zotero.org/google-docs/?C8mEVL) |
| wgbs_classifier_result | (training data only) Cancer signal detection from WGBS assay and classifier [26] |
| cfdna_conc_ng_ml | (training data only) cell-free DNA (cfDNA) concentration in plasma in ng / ml |

A value is set to NA for numerical values and "Non-informative" or "Other/missing" for categorical values if the information could not be determined from clinical data, pathology and radiology reports submitted to the study EDC.

### **Analysis code**

The supplemental data tables and code written in R to generate the tables, lists, and figures shown in the manuscript are available at https://github.com/grailbio-publications/Bredno_PLOS_2021.
